# Simultaneously Boosting Freshwater Productivity and Antifouling Efficacy of Membrane Distillation Through In Situ Micro‐Bubble Generation

**DOI:** 10.1002/advs.202507246

**Published:** 2025-09-29

**Authors:** Zhongsheng Li, Faheem Hassan Akhtar, Xin Cui, Xiaoxiong Wang, Qian Chen

**Affiliations:** ^1^ Shenzhen Key Laboratory of Advanced Technology for Marine Ecology Tsinghua University Shenzhen 518055 China; ^2^ Institute for Ocean Engineering Tsinghua Shenzhen International Graduate School Tsinghua University Shenzhen 518055 China; ^3^ Department of Chemistry and Chemical Engineering Lahore University of Management Sciences (LUMS) Lahore 54792 Pakistan; ^4^ Institute of Building Environment and Sustainable Technology Xi'an Jiaotong University Xi'an 710049 China

**Keywords:** flux enhancement, hypersaline wastewater, membrane distillation, micro‐bubbles, ultra‐antifouling

## Abstract

Membrane distillation (MD), combining phase‐change purification with membrane separation, is a promising technology for treating industrial hypersaline wastewater with complex organics and residual oxidants (e.g., H_2_O_2_). However, membrane fouling remains a prominent challenge that limits both productivity and durability. To address this challenge, a superhydrophobic membrane capable of in situ micro‐bubble generation is developed at the membrane‐liquid interface, effectively mitigating salt accumulation while enhancing vapor transfer. This is achieved by applying a surface coating of γ‐MnO_2_ and perfluorodecyltrichlorosilane (FDTS) on a commercial polyvinylidene difluoride (PVDF) membrane, which facilitates the decomposition of H_2_O_2_ in the feed solution. The modified membrane evinced a flux enhancement of up to 35% for desalinating sodium chloride (NaCl) solution under various operating conditions, and its resistance to gypsum (CaSO_4_) fouling nearly doubled compared to the unmodified membrane. These improvements are attributed to the synergistic effects of the superhydrophobic property and the dynamic micro‐bubbles, which intensified turbulence and acted as nucleation barriers. Compared to recent studies, the developed membrane demonstrated superior productivity, antifouling, and cost‐effectiveness across various scenarios. The work provides a scalable and efficient approach for MD applications in hypersaline wastewater treatment.

## Graphical abstract

A superhydrophobic functional coating composed of γ‐MnO_2_ and perfluorodecyltrichlorosilane (FDTS) is capable of decomposing H_2_O_2_ and generating in situ micro‐bubbles on the membrane surface. The dynamic bubbles intensify the turbulence, enhancing the heat and mass transfer during membrane distillation (MD). This innovative approach demonstrates superior productivity, antifouling properties, and cost‐effectiveness in MD application scenarios compared to previous studies.

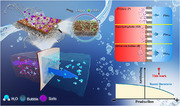

## 1 Introduction

Hypersaline wastewater generated from industrial processes, such as electroplating, textile printing/dyeing, and pharmaceutical manufacturing, constitutes a recalcitrant wastewater category characterized by the dual challenges of salinity inhibition and complex usage residues (e.g., organics, oxidants).^[^ ^1^, ^2^ ^]^ Conventional robust treatment approaches, such as advanced oxidation processes and catalytic ozonation,^[^ ^3^ ^]^ exhibit significantly reduced efficacy in hypersaline matrices. Moreover, the treatment byproducts and residual oxidants (e.g., H_2_O_2_) exacerbate environmental pollution and necessitate secondary treatment.^[^ ^4^ ^]^ In this context, membrane distillation (MD) emerges as an innovative technology that complements hypersaline wastewater treatment approaches.^[^ ^5^ ^]^ As a thermally‐driven hybrid process combining vapor‐pressure gradients and selective phase transitions,^[^ ^6^ ^]^ MD achieves >99.9% salt rejection while effectively removing organic contaminants, even in brines exceeding 50 g L^−1^ TDS, where reverse osmosis (RO) becomes impractical.^[^ ^7^ ^]^ By integrating a crystallizer in the downstream, MD even allows complete separation of salt and water, thus achieving zero‐liquid discharge.^[^ ^8^ ^]^ MD also demonstrates high sustainability through its ability to utilize low‐grade thermal sources (45–80 °C), such as solar heat, geothermal energy and waste heat.

Despite its inherent advantages, MD inevitably suffers from salt scaling (or fouling) on the membrane surface during the evaporation process, leading to pore wetting and ultimately degrading the performance in terms of freshwater productivity, salt rejection and membrane durability.^[^ ^9^ ^]^ To address this challenge, many studies have focused on improving anti‐scaling or anti‐fouling properties of membranes.^[^ ^10^ ^]^ One common approach involves increasing membrane surface roughness through grafting fluorinated coatings, which enhance hydrophobicity and create a barrier to salt nucleation.^[^ ^11^ ^]^ However, such coatings may also lead to pore clogging and flux reduction.^[^ ^12^ ^]^ Another method is the application of photocatalytic^[^ ^13^ ^]^ or electrochemical^[^ ^14^ ^]^ materials on membranes to degrade organic pollutants or to electrostatically repel salts. While these techniques improve fouling resistance, they are less effective at mitigating inorganic scaling and require additional energy inputs (e.g., light or electricity). This results in higher operating costs and more complex module designs, limiting the practical applications. Alternatively, introducing micro‐bubbles into saline water has shown promise in enhancing turbulence, thereby reducing fouling and increasing flux.^[^ ^15^, ^16^ ^]^ Nevertheless, since the micro‐bubbles are usually generated in the bulk feed water rather than directly at the membrane surface, its potential for resisting and migration fouling has not been fully harnessed.^[^ ^17^ ^]^ Moreover, the existing ways of micro‐bubble generation, such as vortex generators, demand considerable energy consumption and capital investment,^[^ ^18^ ^]^ which further restrict the application of this technology. Consequently, it is critical to develop a more efficient and cost‐effective innovative method to maximize the benefits of micro‐bubbles in MD processes.

**Figure 1:**
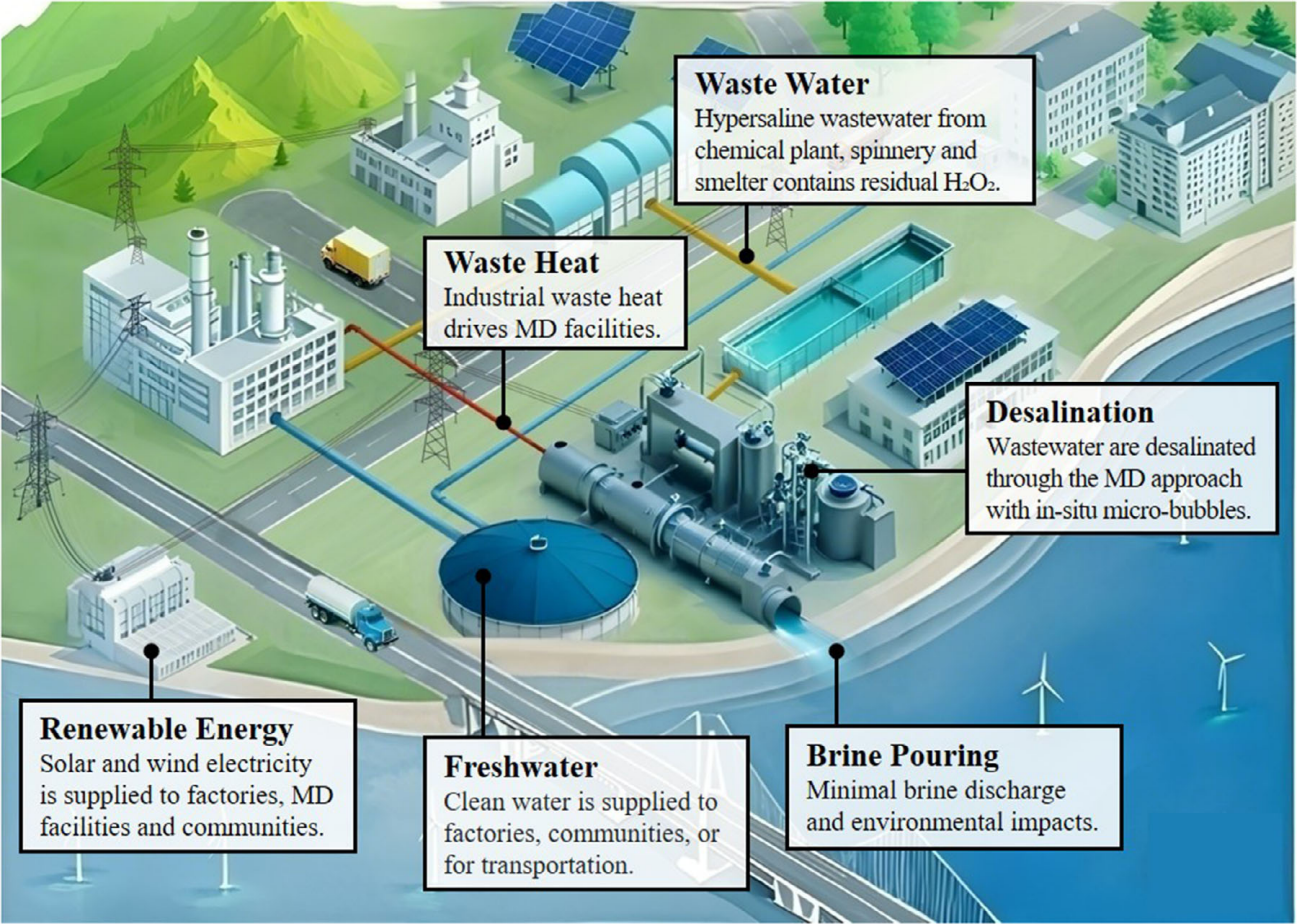
The conceptual MD application for hypersaline wastewater treatment.

Currently, the catalytic decomposition of H_2_O_2_ by metal oxides (e.g., MnO_2_, Fe_2_O_3_) is widely recognized as a stable method for persistently and promptly generating micro‐bubbles at the material contact interface.^[^ ^19^ ^]^ Moreover, with the widespread industrial use of H_2_O_2_ as a versatile reagent for synthesis, bleaching, and oxidation (e.g., chemical plant, spinnery, smelter), the effective recovery of its residues is essential for reducing the consumption losses and minimizing the detrimental environmental impacts.^[^ ^20^ ^]^ Therefore, a new concept for hypersaline wastewater treatment is proposed in this study (as illustrated in **Figure** **1**) to fully reuse residual H_2_O_2_ to continuously generate micro‐bubbles and enhance MD performance. Initially, the wastewater from industries (e.g., chemical plant, spinnery, smelter) contains H_2_O_2_, with residual concentrations typically ranging from 0.1% to 1%,^[^ ^21^, ^22^ ^]^ and undergoes pretreatment in other facilities. Meanwhile, colloids, microorganisms, and specific chemical substances are partially removed.^[^ ^23^ ^]^ Subsequently, the pretreated water is deeply purified with the microbubble‐assisted MD approach. Here, the water can be heated directly by renewable energy and the waste heat from industrial production,^[^ ^24^ ^]^ making this process more practical and sustainable. Finally, the freshwater is collected for domestic use or recycled for industrial production.

A catalytic membrane capable of degrading H_2_O_2_ to generate in situ micro‐bubbles is critical for the proposed MD treatment approach. In this study, we fabricate the conceptual membrane by coating a commercial polyvinylidene difluoride (PVDF) substrate with γ‐MnO_2_ and perfluorodecyltrichlorosilane (FDTS), enabling the decomposition of H_2_O_2_ in the feed solution. The resulting in situ micro‐bubbles enhance turbulence and minimize salt accumulation on the membrane surface, leading to increased productivity and improved antifouling performance. We conduct quantitative analyses over the effect of the in situ micro‐bubbles in DCMD process, and the underlying mechanisms for the observed improvements are revealed experimentally and theoretically. Additionally, the application potential and feasibility of the developed method in practical scenarios are discussed, considering both environmental and financial factors. Our work demonstrates an innovative separation membrane specifically tailored for the treatment of hypersaline organic‐laden wastewater, offering new possibilities for advancing membrane distillation processes.

## 2 Results and Discussion

### 2.1 Fabrication and Characterization of Novel Membranes

**Figure 2:**
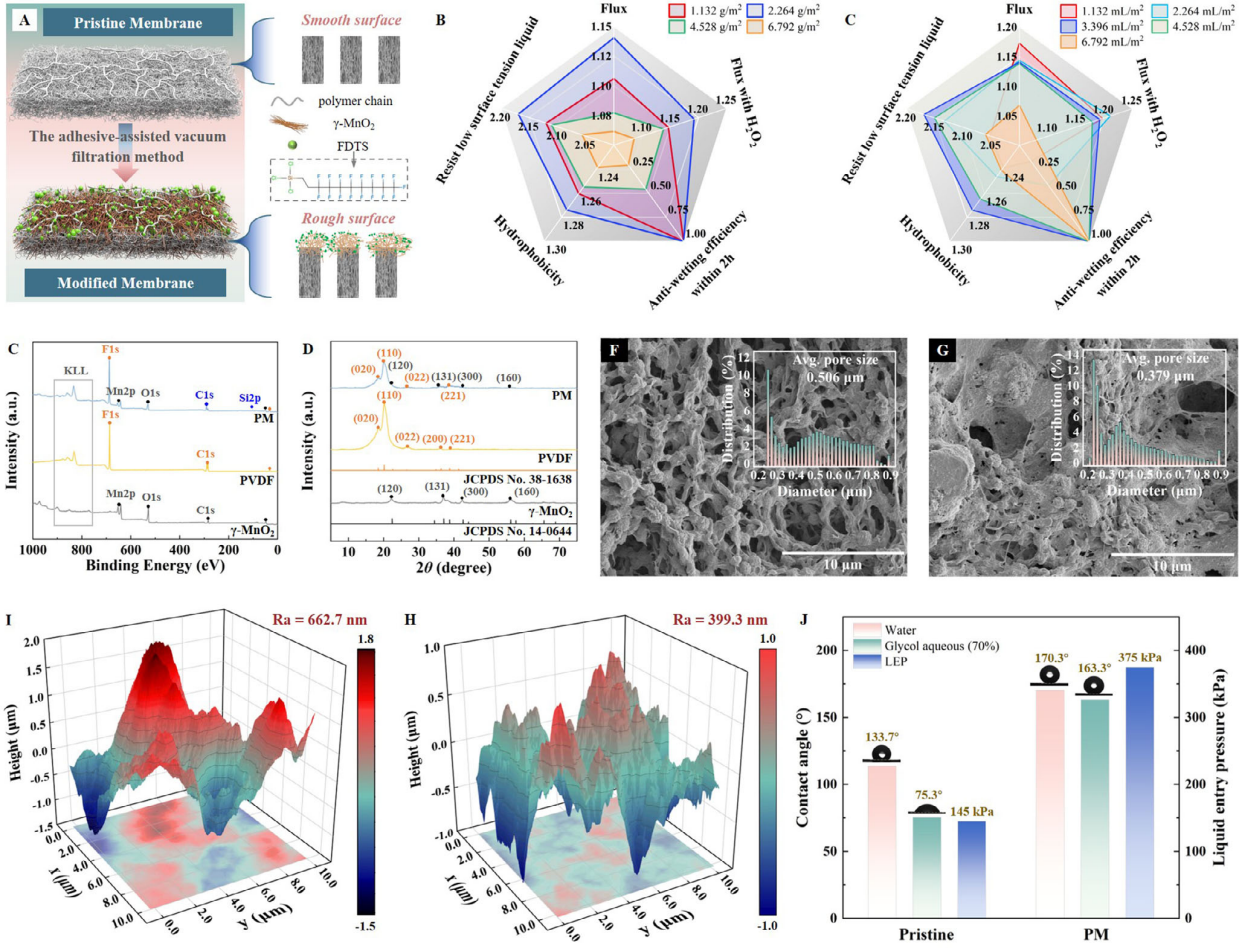
**Optimum fabrication parameters and membrane characterization**. (A) Formation schematic of the modified membrane. Normalized comparison of membrane properties with different proportions of (B) γ‐MnO_2_ and (C) perfluorodecyltrichlorosilane (FDTS). (D) XRD patterns. (E) XPS survey spectra. Surface morphology of (F) the pristine membrane and (G) the PM. Average Ra values of (H) the pristine membrane and (I) the PM. (J) Water contact angle, glycol aqueous solution contact angle and LEP of the membranes.

The high‐performance membrane with in situ micro‐bubbles was fabricated by coating the functionalized superhydrophobic layer onto a commercial PVDF membrane, as shown in **Figure** **2**A. Optimizing the fabrication scheme is crucial for balancing high‐flux and antifouling properties, as the loading amount of the functional materials (γ‐MnO_2_ and perfluorodecyltrichlorosilane (FDTS)) directly impacts the pore structure and surface characteristics.^[^ ^25^, ^26^ ^]^ To quantify the effects of optimization, the performance variables of the pristine membrane are represented as a benchmark, and the membrane properties are compared using a normalized method (the experimental setup and analytical procedures are provided in Sections S1 & S2, Supporting Information).

With increasing γ‐MnO_2_ content, all performance variables initially increase followed by a decrease (Figure 2B). Optimal values are achieved at a γ‐MnO_2_ content of 2.264 g m^−2^. Notably, compared with those of the pristine membrane, the flux and flux with H_2_O_2_ (with micro‐bubbles) increase by 6.8% and 17.2%, respectively. Moreover, no flux reduction is observed during SDS solution filtration within 2 h, and the glycol aqueous contact angle reaches 163.3°, indicating robust superhydrophobicity. On the other hand, higher FDTS dosage induces divergent performance variables (Figure 2C), the hydrophobicity and antiwetting (antifouling) efficiency peak at 3.396 mL m^−2^, whereas the highest production occurs at a lower 1.132 mL m^−2^. This is because more intense fluoridation increases the surface roughness, yet excessive grafting obstructs pores and reduces flux. Specifically, the maximum flux improvement is only 13.2%, while the wettability variation exceeds 82.9%. The corresponding experimental results are detailed in Section S3 (Supporting Information). Consequently, balancing high flux and antifouling, the optimal composite membrane (denoted PM) is prepared using 2.264 g m^−2^ γ‐MnO_2_ and 3.396 mL m^−2^ FDTS.

Further, the chemical structures were analyzed via XRD (Figure 2D) and XPS (Figure 2E) to investigate the effects of the fabricated materials on pore structure and surface characteristics. The results reveal new peaks corresponding to Mn, O, and Si in the PM, confirming that a mixture of γ‐MnO_2_ and FDTS was successfully coated without altering original properties (the detailed analyses are given in Section S4, Supporting Information). The network topography of both the pristine membrane and the PM are given in Figure 2F and Figure 2G, respectively, with the corresponding parameters summarized in **Table** **1**. It is evident that the pore size of the PM is smaller than that of the pristine membrane, which results from the γ‐MnO_2_ nanofilaments are tightly adhered on the membrane surface by Nafion molecules. Despite the reduction in pore size, the flux of the PM has increased. This increase is attributed to the rough surface created by the grafted fluoride (numerous granular protrusions), which improves the evaporation contact line and enhances fouling resistance.^[^ ^27^ ^]^

**Table 1:** Details of the parameters of related to the membrane characteristics.

Membrane	Thickness [mm]	Average pore size [µm]	Roughness [nm]	Porosity	Tortuosity
Pristine	0.12	0.506	399.3	0.698	1.441
PM	0.13	0.379	662.7	0.606	1.545

The critical metrics for antifouling evaluation, surface roughness and hydrophobicity, are assessed through AFM and contact angle measurements.^[^ ^28^, ^29^ ^]^ The pristine membrane has a smooth surface with an Ra value of 399.3 nm (Figure 2H), whereas the PM has a significantly increased Ra value of 662.7 nm (Figure 2I). In general, a rougher surface exhibits improved antifouling properties, primarily due to the “air pockets” that expand the air‐liquid contact area and stabilize the Cassie‐Baxter state.^[^ ^30^, ^31^ ^]^ As shown in Figure 2J, the PM demonstrates markedly larger water and glycol aqueous contact angles (170.3° and 163.3°) compared to the pristine membrane (133.7° and 75.3°). Furthermore, the liquid entry pressure (LEP) of the PM doubles to 380 kPa, indicating robust resistance to liquid intrusion (the LEP measurement method and comparative results with recently reported modified membranes are provided in Section S5, Supporting Information). The concurrent enhancements in roughness and hydrophobicity suggest the superior antifouling performance of the PM, which will be confirmed in detail in the subsequent sections.

### 2.2 Production Performance of DCMD

**Figure 3:**
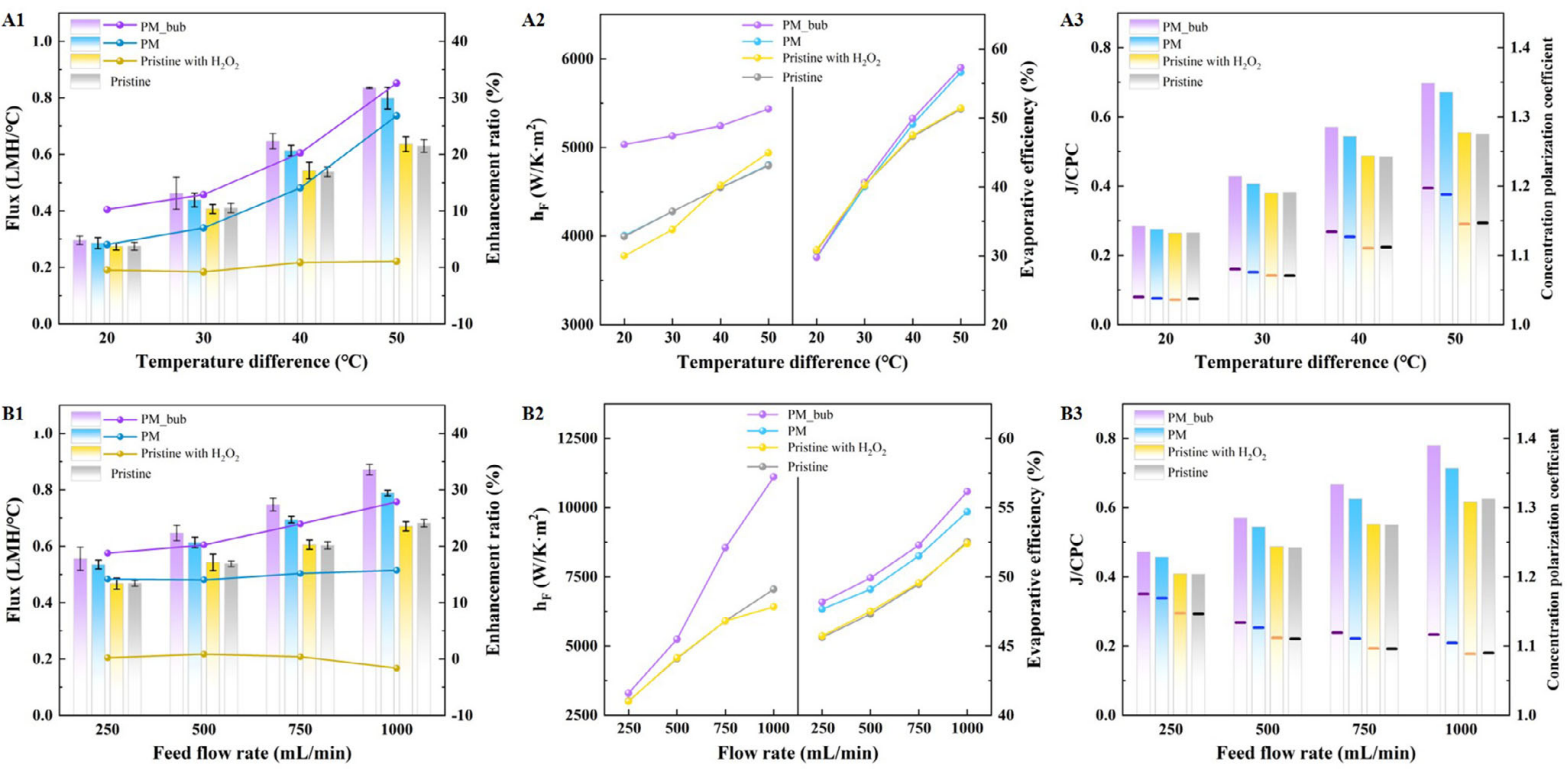
**Production performance for desalinating sodium chloride solution under various operating conditions**. (A1) The flux and enhancement ratio, (A2) the heat transfer coefficient and evaporative efficiency, and (A3) the *J/CPC* (column) and *CPC* (line) at different *ΔT* with a flow rate of 500 mL min^−1^. (B1 – B3) The corresponding results for different flow rates at a *ΔT* of 40 °C.

To highlight the potential of the modified membrane (PM) for enhancing productivity, we performed DCMD tests under various operating conditions (temperature difference and feed flow rate). The production performance at different *ΔT* (temperature difference between feed and permeate sides) with a flow rate of 500 mL min^−1^ is shown in **Figure** **3A1**. The fluxes of all the treatment groups increase significantly with increasing temperature, and the PM exhibits higher flux than the pristine membrane. This is because the driving force for water permeation is the vapor pressure gradient across the membrane, which exponentially increases with *ΔT*. When *ΔT* increases from 20 °C to 50 °C, the flux‐enhancement ratio of the PM over the pristine membrane increases from 4.05% to 26.83%. Notably, the production enhancement of the PM with micro‐bubbles (PM_bub) further increases by ≈6%, with the addition of H_2_O_2_ to the feed fluid. These results indicate that the PM can significantly improve productivity in the DCMD process.

Numerical simulations were conducted to reveal the vapor permeability and concentration accumulation on the membrane surface via heat and mass transfer theory (the model formulation procedure is provided in Sections S6–S10, Supporting Information). With the implementation of the established mathematical model, the actual heat transfer coefficient (*h_F_*) and concentration polarization coefficient (*CPC*) on the feed side were calculated. As *ΔT* increases, the *h_F_* and evaporation efficiencies of both the pristine membrane and the PM also increase (Figure 3A2). When H_2_O_2_ decomposes and generates micro‐bubbles on the PM surface, there is a substantial increase in the *h_F_*, corresponding to the flux enhancement. The most significant improvement (≈25%) is observed at the lowest temperature difference (*ΔT* = 20 °C). Generally, low operating temperature differences lead to a thick boundary layer and increase bubble formation.^[^ ^32^ ^]^ Concurrently, high fluid viscosity with greater internal shear stress facilitates bubble collapse and intensifies eddies,^[^ ^33^ ^]^ which amplifies the micro‐bubble enhancement. Furthermore, despite the higher *CPC* compared with the other three groups, the PM_bub still exhibited the highest flux under an equivalent *CPC*, as evidenced by the *J/CPC* ratio depicted in Figure 3A3. This observation provides additional confirmation that the in situ micro‐bubbles effectively prevent the concentration accumulation and maintain stable permeation.

The feed flow rate also plays an important role in the production performance of the DCMD process. The results obtained at different flow rates with a *ΔT* of 40 °C are presented in Figure 3B1. As is well known, a higher flow rate induces greater turbulence, resulting in lower heat and mass transfer resistance.^[^ ^34^, ^35^ ^]^ Thus, the fluxes of all the groups increase with the flow rate. Under all flow rates, the PM_bub consistently exhibits the highest flux, whereas the pristine membrane exhibits the lowest flux. Moreover, the flux‐enhancement ratio of the PM_bub over the pristine membrane clearly increases with increasing flow rate, ranging from 18.8% to 27.8%. In contrast, the enhancement ratio of the PM remains constant at ≈15% in the absence of H_2_O_2_. As illustrated in Figure 3B2, the impact of the flow rate on *h_F_* is more substantial than that of the temperature, and the enhancement becomes more pronounced with increasing flow rate. This is attributed to the intensified turbulence on the feed side as the flow rate increases, which reduces the boundary layer thickness.^[^ ^15^, ^17^ ^]^ When the flow rate increases to 1000 mL min^−1^, the *h_F_* of the PM_bub reaches a peak value of 11 106.38 W m^−2^·K^−1^, whereas the *h_F_* of the pristine membrane is only 7050.16 W m^−2^·K^−1^. Furthermore, the *CPC* decreases with increasing flow rate, and the PM_bub consistently has the highest *J/CPC* ratio (Figure 3B3). These phenomena can be explained by the dynamic activities of the bubbles becoming more intense at high flow rates, which notably enhances the heat and mass transfer on the feed side. In conclusion, the modified membrane with in situ micro‐bubbles generated by decomposing H_2_O_2_ demonstrated remarkable productivity enhancement under various operating conditions.

As the temperature, velocity and concentration fields on the feed side are changed when micro‐bubbles occur, existing empirical correlations become insufficient for accurately predicting the heat transfer coefficients.^[^ ^17^, ^36^ ^]^ Therefore, the correlation must be modified to improve the predictive capabilities of the model. A new correlation for predicting the Nusselt number under micro‐bubble enhancement is developed based on the experimental data (the detailed procedure is provided in Section S9, Supporting Information). The new Nusselt correlation is given in Equation (1).

(1) $$Nu=0.0123Re^{1.042}\cdot Pr^{1.029}$$

This modified correlation remarkably enhances the model prediction accuracy for DCMD process, and detailed model verification with experimental data is presented in Section S11 (Supporting Information).

### 2.3 Antifouling Performance of DCMD

**Figure 4:**
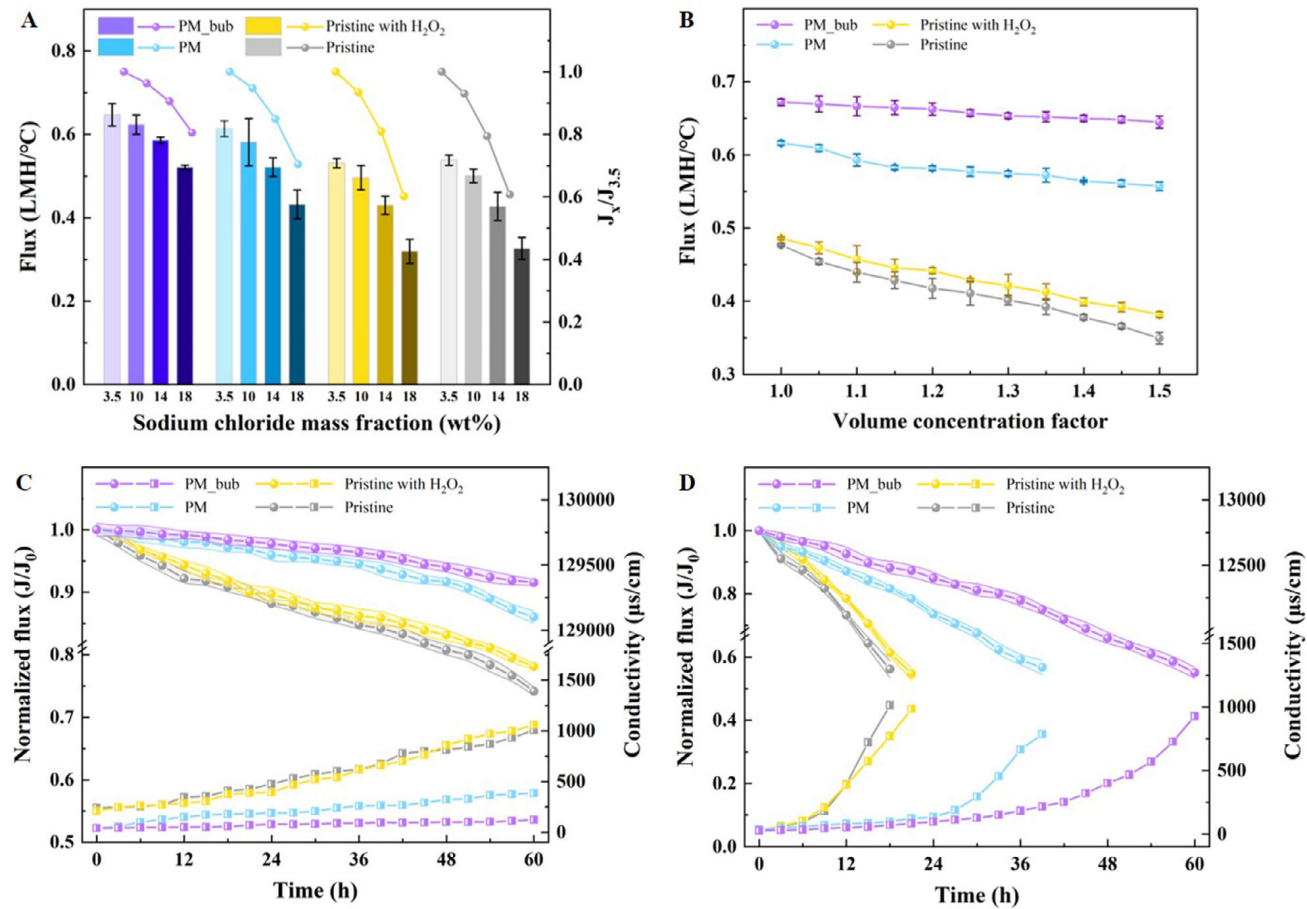
**Antifouling performance and long‐term durability of the treatment groups**. (A) Flux and flux variation *Jx/J_3.5_* (ratio of flux at NaCl concentration of x wt% to flux at 3.5 wt%) at different NaCl mass fractions. (B) The flux variation at different volume concentration factors (VCFs) with the initial CaSO_4_ supersaturated solution (≈5 g L^−1^). Long‐term desalination for (C) NaCl (10 wt%) and (D) gypsum solution (≈5 g L^−1^).

For DCMD desalination, scaling and fouling evolution ​is strongly dependent on​ the properties of the feed solution. Typically, the primary types of scales are categorized as highly soluble salt scales (NaCl) and sparingly soluble salt scales (CaSO_4_). The experimental results from treating NaCl solutions with different mass fractions (3.5 – 18 wt.%) demonstrate that the flux decreases as the NaCl concentration increases in all the groups (**Figure** **4**A). This reduction in flux is due to a decrease in partial vapor pressure resulting from the increased salt content.^[^ ^37^ ^]^ Through the evaluation of flux susceptibility to NaCl concentration, the observed trend in *Jx/J_3.5_* (ratio of flux at salinity of x wt% to flux at 3.5 wt%) indicates that higher salinity leads to greater flux reduction. Specifically, the PM_bub consistently exhibits the lowest flux reduction across the concentration gradient, which is because the in situ micro‐bubbles effectively inhibit salt accumulation on the membrane surface.

During the gypsum scaling process (≈8 h), no significant decrease in rejection rate (> 99%) was observed in all four groups. Thus, the decline in flux is utilized to infer the scaling propensity of the membrane. As illustrated in Figure 4B, the PM shows better anti‐scaling performance than the pristine membrane, which is evidenced by the volume concentration factor (VCF) when the normalized flux reduction occurs. The PM is still susceptible to gypsum, leading to a decrease in flux when VCF exceeds 1.25. In contrast, with micro‐bubbles generated on the PM surface, there is no flux reduction even when the feed solution is concentrated by 1.5‐fold. Moreover, its initial flux (0.672 LMH °C^−1^) for desalinating gypsum solution is enhanced by 40% compared to the pristine membrane (0.477 LMH °C^−1^). By observing the cross‐sectional SEM images and EDS maps of the membrane surface before and after the experiments (the detailed analysis is given in Section S12, Supporting Information), heavy “needle‐like” scaling with an average thickness of 120 µm is evident on the pristine membrane. In contrast, the scaling thickness and Ca element detected on the PM_bub surface are nearly negligible.

To investigate membrane stability under long‐term DCMD operation, the continuous 60‐h desalination experiments were conducted. As shown in Figure 4C&4D, the flux gradually declines with conductivity increases, indicating progressive membrane scaling. Initially, all treatment groups maintained NaCl rejection rates exceeding 99.8% for the first 10 h. With prolonged operation, the rejection rate of the pristine membrane rapidly decreased below 99.5%, accompanied by a significant drop in flux to 75% of the initial flux after 60 h. In contrast,​​ the modified membrane (PM) consistently maintained rejection rates exceeding 99.5% throughout this period. Notably, the PM_bub exhibited exceptional stability, retaining over 99.8% rejection with only an ≈8% flux decline. Performance deterioration intensified when challenged with a scaling‐prone supersaturated gypsum solution. The rejection rate of the pristine membrane fell to 98% within 12 h ​and​ plunged below 95% in the next 3 h, with normalized flux also decreasing to 0.6. Although the PM extended operating durability, significant reductions in both rejection and flux still occurred after 36 h. However, the PM_bub demonstrated a markedly antifouling advantage, exhibiting a mitigated performance degradation. Despite its rejection rate eventually approaching a critical value after continuous operation (≈57 h), the PM_bub sustained effective desalination for 4 times and 1.5 times longer than the pristine membrane and the standard hydrophobic modified membrane (PM), respectively. This outstanding scaling resistance is primarily attributed to the dynamic behaviors of the in situ generated bubbles that alleviate scaling. Additionally, the membrane intrinsic stability was assessed through​ coating material leaching detection. Minor loss of inadequately bonded materials was observed, yet this detachment caused no observable performance impairment (the inductively coupled plasma (ICP) analysis results are provided in Section S13, Supporting Information), further validating the long‐term stability. Collectively, these findings establish the significantly superior antifouling performance of the PM_bub relative to the conventional DCMD process and confirm its enhanced long‐term durability.​

### 2.4 Mechanisms for Improving Production and Antifouling Performance

Most of the modified superhydrophobic MD membranes with higher fouling resistance typically exhibit lower fluxes^[^ ^38^, ^39^ ^]^ (literature reviews are discussed in the subsequent section). However, as demonstrated by previous tests, the modified membrane (the PM) in this work clearly exhibits simultaneous improvement in both production and antifouling performance. Therefore, elucidating the underlying mechanisms responsible for these improvements is crucial. To facilitate a clearer and more comprehensive analysis, the mechanisms contributing to production and antifouling enhancements are explained in the two distinct aspects.

**Figure 5:**
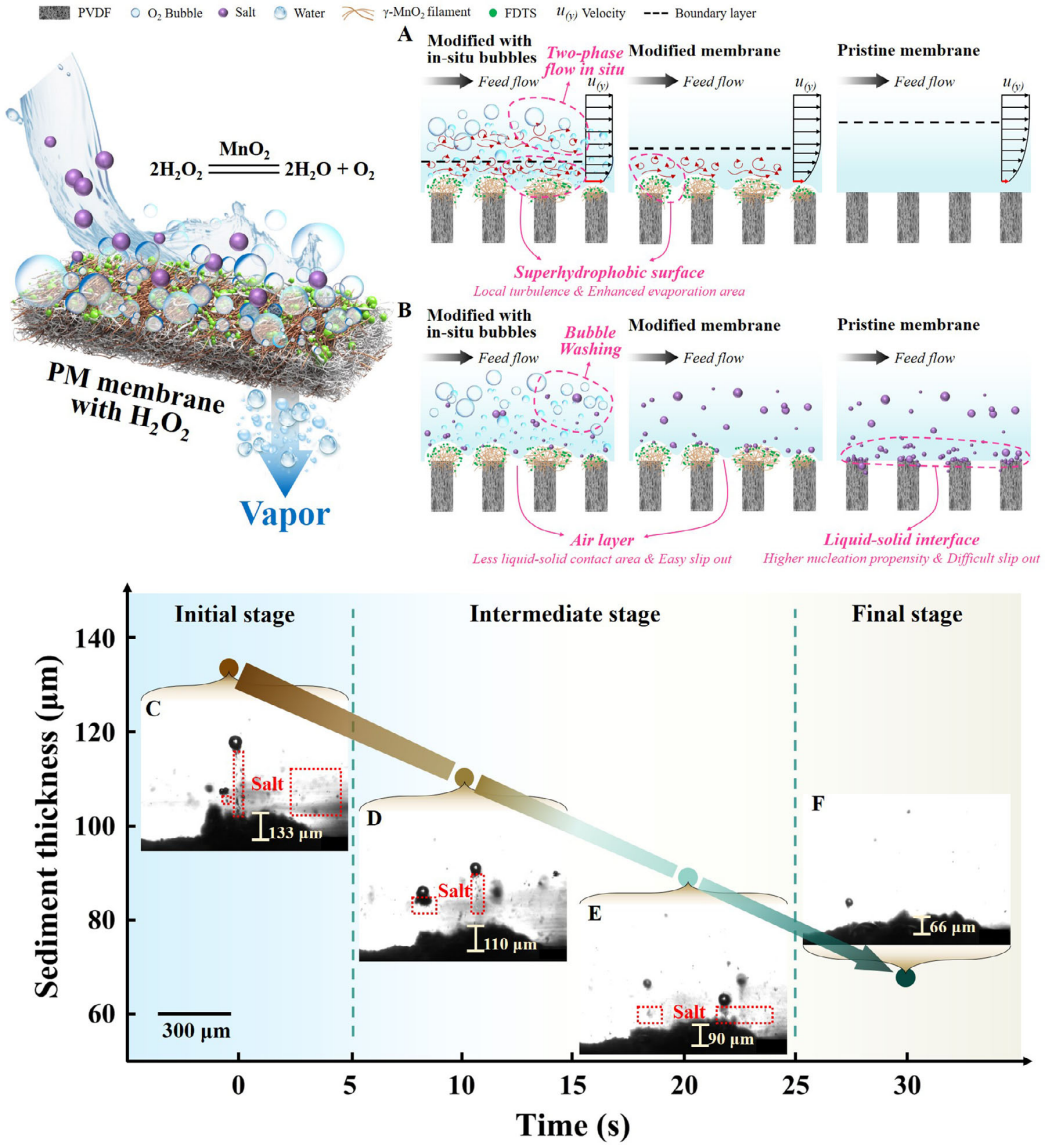
**Schematic of the mechanism analysis**. Mechanistic information of the enhanced (A) production performance and (B) antifouling performance. (C) The initial stage (0–5s), (D, E) intermediate stage (5–25s) and (F) final stage (25–30s) of the salt removal process by in situ micro‐bubbles.

The first mechanism for flux enhancement is attributed to the larger effective evaporation area of the PM (**Figure** **5**A). Owing to its superhydrophobic surface, the PM maintains a contracted contact line (or a larger contact area) with the solution, serving as the theoretical evaporation surface. The second mechanism is related to changes in the boundary layer above the membrane surface. Nanoscale eddies generated by the nanowires and fluoride particles loaded on the membrane surface alter the flow pattern at the solid‐liquid contact interface, reducing the boundary layer thickness. These two aforementioned mechanisms have been supported by many previous studies.^[^ ^27^, ^40^, ^41^ ^]^ Additionally, the third mechanism is specific to the PM_bub, where the generated bubbles enhance the turbulence on the feed side (as recorded in Video S1, Supporting Information), thereby further reducing the boundary layer and promoting heat and mass transfer. This is the primary reason for the higher flux observed in the in situ micro‐bubble enhanced DCMD process.

Membrane fouling is primarily caused by the process of salt precipitation, which is exacerbated by foreign particulates.^[^ ^42^ ^]^ In addition, nucleation crystallization occurs only when the local residence time exceeds the nucleation time. Therefore, fouling can be reduced by decreasing the propensity for nucleation or shortening the residence time. According to the Gibbs free energy barrier law (Section S14, Supporting Information), an increase in the liquid‐solid contact angle corresponds to a greater nucleation barrier, increasing the difficulty of crystallization.^[^ ^11^ ^]^ It is widely acknowledged that a membrane with a superhydrophobic surface has a larger contact angle and air‐liquid contact interface^[^ ^43^, ^44^ ^]^ (Figure 5B). This results in the formation of a “slip layer”, which reduces salt adhesion and consequently shortens the residence time on the membrane surface. Nevertheless, as discussed in the previous section, salt crystals are still observed on the superhydrophobic surface of the PM, although the anti‐scaling ability is improved. Excitingly, the in situ generated bubbles effectively remove the salts precipitated on the membrane surface, which is recorded via a high‐speed tracking camera (the recording process is provided in Section S15, Supporting Information). The membrane surface was initially deposited with a layer of gypsum, and bubbles (10–200 µm) were generated on the membrane surface during H_2_O_2_ decomposition (as demonstrated in Video S2, Supporting Information). In the initial stage (Figure 5C), “splashed” salts are observed as the bubbles move upward, indicating that the bubbles help to disperse the precipitated salts. Subsequently, the micro‐bubbles carry the salts away, with the salts adhering to the bubbles (Figure 5D and Figure 5E). As a result, “air scouring” effectively removes salts, as evidenced by the reduction in scaling thickness from 133 to 66 µm within 30 s (Figure 5F).

### 2.5 Literature Comparison and Application Feasibility

**Figure 6:**
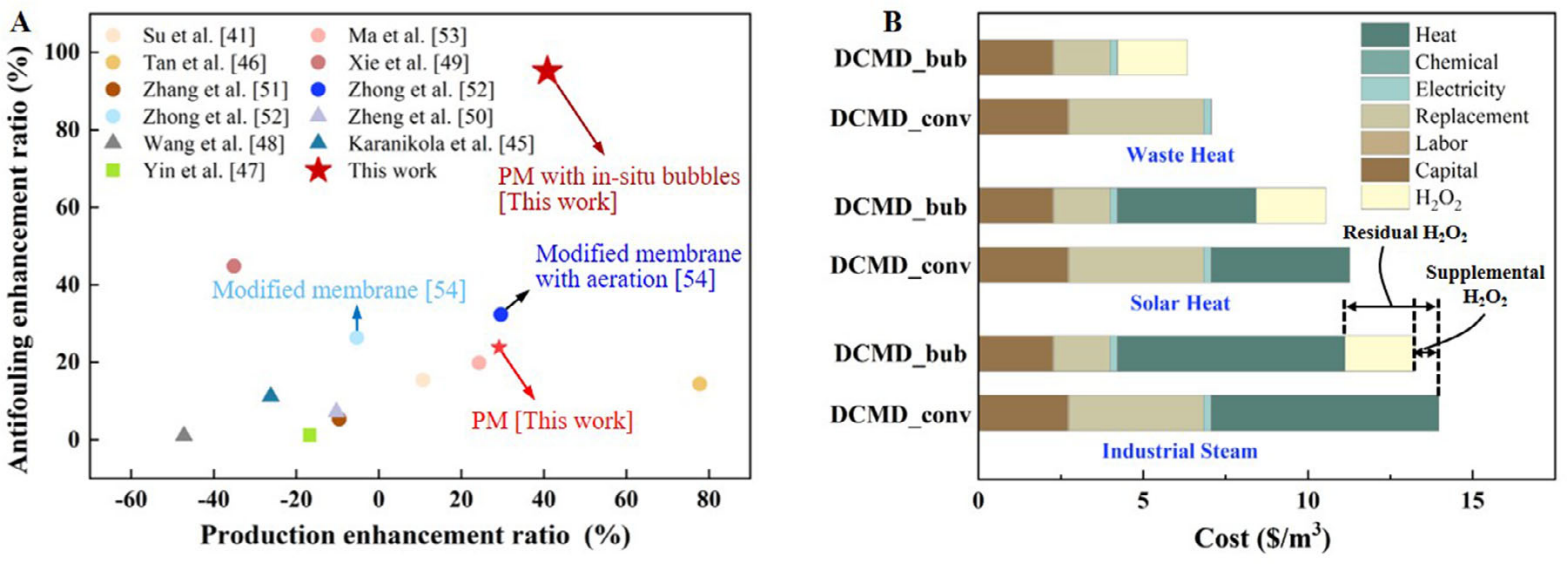
**Literature comparison and cost evaluation**. (A) Comparison of production and antifouling performance, where the gypsum concentrations of the circles (○), triangles (△), rectangles (⬜) and stars (☆) were 2 g L^−1^, 2.7 g L^−1^, 2.4 g L^−1^ and 5 g L^−1^, respectively. (B) The UPC estimation of conventional DCMD_conv and novel DCMD_bub approach in pilot‐scale applications under waste heat (0 $ kWh^−1^), solar heat (0.028 $ kWh^−1^) and industrial steam (34.5 $ ton^−1^)).

The properties of the PM are summarized and compared with those of typical studies on the DCMD process conducted within the past 5 years,^[^ ^41^, ^45^, ^46^, ^47^, ^48^, ^49^, ^50^, ^51^, ^52^, ^53^ ^]^ highlighting the superiority of the modified membrane presented in this work (**Figure** **6**A; Section S16, Supporting Information). The most commonly used method for superhydrophobic membrane modification is dip coating, which has been reported to improve the antifouling performance up to 78%. However, enhancing production through this preparation method is challenging because of the pore blockage and porosity reduction. Consequently, in more than half of the previous studies, the modified membranes exhibit a lower flux compared to the pristine membrane. Notably, the study conducted by Zhong et al.^[^ ^52^ ^]^ reported the preparation of superhydrophobic membranes and the investigation of the effects of aeration on flux and antifouling performance, similar to our research process. The results indicated that aeration assistance increased the flux, but its impact on improving fouling resistance was insignificant. In this work, the modified superhydrophobic membrane (the PM) demonstrates a higher flux and lower scaling propensity simultaneously during the treatment of a hyperconcentrated gypsum solution (≈5 g L^−1^). Furthermore, the PM with micro‐bubbles exhibits a top‐tier antifouling performance with an impressive flux increase of over 40% compared with that of the pristine membrane.

We also conducted the cost evaluation of the proposed novel DCMD with in situ bubbles (DCMD_bub) in a pilot‐scale application (24 m^3^ d^−1^). The analysis procedure and detailed results are presented in Section S17 (Supporting Information). Since dip‐coating is technically straightforward and requires no extra complex equipment, the primary cost for the modified membrane is attributed to the extra material consumption, i.e., MnO_2_, FTDS and Nafion. As shown in Figure 6B, the initial cost of the DCMD module using the modified membrane is increased by only 1.8%. Due to the improvements in productivity and durability, the initial investment and the MD module replacement cost are reduced by 17% and 58%, respectively. Furthermore, the Unit Production Cost (UCP) analysis across three common low‐grade heat sources indicates the economic superiority of the DCMD_bub approach under declining heat prices. Compared to the conventional DCMD (DCMD_conv), the UPC shows reductions of 20%, 25% and 40% when powered by industrial steam, solar heat and waste heat, respectively. Remarkably, as previously described in the conceptual application of DCMD_bub, the residual H_2_O_2_ concentrations (0.1–1%) are sufficient to drive the process. Even in extreme scenarios requiring supplemental H_2_O_2_, this approach still offers significant cost advantages, reducing the UPC by ≈10%. In conclusion, MD process with in situ micro‐bubbles developed in this work represents a promising avenue for environmentally, technologically and economically water treatment.

## 3 Conclusions and Implications

In the current work, we have successfully achieved simultaneous high flux and fouling resistance in the MD process through in situ micro‐bubble generation on a novel membrane. Compared to the pristine membrane, the surface characteristics of the modified membrane (PM) were significantly improved (e. g., Ra and LEP doubled as 662.7 nm and 375 kPa), suggesting its superior hydrophobicity. Proof‐of‐concept experiments found that with in situ micro‐bubbles generated, the membrane (PM_bub) achieved a flux enhancement of 10–35% for desalinating sodium chloride solution under various operating conditions (including different temperatures, fluid flow rates and salt concentrations). Moreover, the rejection rates and durability were significantly enhanced during the long‐term desalination process, with stable operation against gypsum scaling extended to 60 h (compared to only 12 h for conventional membranes), indicating its exceptional antifouling performance. These enhancements were further validated and quantified through calculated heat transfer and concentration polarization coefficients by a numerical model. The key mechanism driving the robust performances is that the superhydrophobic coating and in situ micro‐bubbles synergistically enhanced turbulence and nucleation barriers on the membrane surface.

Although the fundamental principles of these improvements have been laid out, the detailed effects of the dynamic behavior of in situ micro‐bubbles on the velocity, temperature, and concentration at the evaporation interface and in the fluid bulk remain unclear. Therefore, in future investigations, comprehensive multi‐physics field computation and visual analysis of this MD process are crucial for refining the underlying mechanisms. In this work, the developed membrane outperformed recent similar studies in both productivity and antifouling performance, and its pilot‐scale application evaluation also demonstrated cost‐effectiveness across various heat sources (UPC decreasing by up to 40%). Based on technological comparisons and feasibility analyses, the primary focus for the future work is to industrially fabricate larger modified membranes and determine the performance in specific application scenarios. Overall, this work introduces a promising membrane design approach and lays the groundwork for broader industrial applications of MD in hypersaline wastewater treatment.

## 4 Method

### Materials

A commercial PVDF membrane (pristine) from Merckmillipore was used. Nafion was purchased from DuPont, and γ‐MnO_2_ was obtained from Dalian Tongmeng New Material Technology Corp, China. Ethanol (C_2_H_6_O), hydrogen peroxide (H_2_O_2_), perfluorodecyltrichlorosilane (FDTS), sodium chloride (NaCl), calcium chloride (CaCl_2_), sodium sulfate (Na_2_SO_4_) and sodium dodecyl sulfate (SDS) were purchased from Macklin. All the chemicals were used directly in the experiments without further purification.

### Membrane Fabrication

The flat‐sheet composite membranes were prepared via the vacuum filtration coating method, and the detailed modification scheme is provided in Section S2 (Supporting Information). The general preparation procedure is as follows: First, γ‐MnO_2_ was added to a 50% ethanol solution, followed by FDTS. The mixed solution was subsequently subjected to ultrasonic treatment in a 35 °C bath for 2 h. Next, Nafion (3.396 mL m^−2^) was introduced into the solution as a binder and stirred for 5 min. Under negative pressure, the mixture was codeposited onto the PVDF membranes (49 mm×40 mm). Finally, the membranes were dried at 100 °C in an oven for 24 h.

### Membrane Characterization

The crystallinity of the membranes was investigated via X‐ray diffraction (XRD, Rigaku Ultima IV). Tests were conducted in the range of 5° to 80° with Cu Kα radiation at 35 kV and 35 mA. X‐ray photoelectron spectroscopy (XPS, FEI Escalab 250) was used to analyze the chemical composition of the membranes, employing Al Kα as the excitation source and achieving an energy resolution of 0.6 eV. Furthermore, high‐resolution scans of the Mn (2p) and C (1s) regions were collected in fixed analyzer transmission mode. The surface and cross‐sectional morphologies were examined via scanning electron microscopy (SEM, Hitachi SU8010), and the elemental distribution was analyzed via energy dispersive X‐ray spectroscopy (EDS, FEI Quanta 250). The pore size was determined via the gas permeability method (GPM, Porometer CFP‐1500AE). The porosity and tortuosity of the membranes were determined via the mercury intrusion porosimetry method (MIP, Micromeritics AutoPore 9500). Atomic force microscopy (AFM, Bruker Dimension Icon) was used to analyze the surface roughness, utilizing a typical scanning area of 10 µm × 10 µm. To improve the accuracy of the surface roughness measurements, Ra values were obtained from three distinct locations on the samples and integrated into a composite dataset for AFM analysis. The hydrophobicity of the membranes was evaluated by the water contact angle using a contact angle goniometer (KRUSS DSA30). In addition, the resistance of the membranes to low surface tension liquids was assessed by measuring the contact angle of the glycol aqueous solution (70%).

### DCMD Performance

A typical direct contact membrane distillation (DCMD) system was utilized to conduct the tests (the equipment setup is provided in Section S1, Supporting Information). To investigate the influence of temperature on production performance, the temperature on the feed side was adjusted from 40 to 70 °C while maintaining a constant temperature of 20 °C on the permeate side, where the flow rate on both sides remained at 500 mL min^−1^. Similarly, to study the effect of flow rate on production performance at a *ΔT* of 40 °C (feed side temperature of 60 °C and permeate side temperature of 20 °C), the flow rate on the feed side was varied from 250 to 1000 mL min^−1^ while maintaining a constant flow rate of 500 mL min^−1^ on the permeate side. Importantly, during the microbubble‐assisted experiments, the initial concentration of H_2_O_2_ was set at 0.1 wt%. This dosage ensured sufficient oxidant availability for sustained catalytic reactions throughout the 2‐h testing period (the catalytic reaction mechanism and brief experimental results of flux under different H_2_O_2_ concentrations are provided in Section S18, Supporting Information).

The above experiments were conducted with a salinity of 3.5 wt% and the flux was evaluated as follows:

(2) $$J_{g}=\frac{\Delta m}{\Delta t\cdot \Delta T\cdot A}$$

where *J_g_* (LMH °C^−1^) is the flux of the treatment groups, *Δm* (kg) is the mass of the product water, *Δt* (≈2 h) is the measurement time required to reach stability, *ΔT (°C)* is the temperature difference across the membrane, and *A* (≈0.001960 m^2^) is the membrane area.

The enhancement ratio (%) was determined by comparing the flux of the treatment groups *(J_g_)* to that of the pristine membrane *(J_p_)* under the identical *ΔT* or flow rate conditions, which was calculated as follows:

(3) $$\mathrm{Enhancement}ratio=\frac{J_{g}}{J_{p}}\times 100$$

where *J_g_* (LMH °C^−1^) is the flux of the treatment groups, and *J_p_* (LMH °C^−1^) is the flux of the pristine membrane.

To quantify the improvement in heat transfer and the resistance to concentration accumulation, a numerical model was established to calculate an accurate heat transfer coefficient (*h_F_*) and concentration polarization coefficient (*CPC*) on the feed side. The model formulation and solution process are provided in Sections S6–S10 (Supporting Information). Specifically, the evaporation efficiency was defined as the ratio of the latent heat (J·h_fg_^*^) to the total heat *(Q_m_)* across the membrane, and was calculated as follows:^[^ ^54^ ^]^

(4) $$\mathrm{Evaporation}\;\mathrm{efficiency}=\frac{J\cdot {h_{\mathrm{fg}}}^{*}}{Q_{m}}\times 100$$

where *J* (kg m^−2^·s^−1^) is the mass flux and h_fg_^*^ (J kg^−1^) is the enthalpy of vaporization.

### The *CPC* was defined as follows

(5) $$\mathrm{Concentration}\;\mathrm{polarization}\;\mathrm{coefficient}\;\left(\mathrm{CPC}\right)=\frac{C_{m}}{C_{b}}$$

where *C_b_* (wt%) is the salt concentration in the bulk solution and *C_m_* (wt%) is the salt concentration on the membrane surface. Concentration polarization occurs due to vapor permeation, which means that the concentration on the membrane surface is greater than in the bulk solution.

To assess the antifouling performance against highly soluble and sparingly soluble salts in different treatment groups, high‐concentration NaCl solutions (3.5 wt%, 10 wt%, 14 wt%, and 18 wt%) and a supersaturated CaSO_4_ solution (5 g L^−1^) of 1000 mL were prepared. The experiments were conducted at a fixed *ΔT* of 40 °C with a flow rate of 500 mL min^−1^. The susceptibility of flux to changes in salinity was utilized to approximately evaluate scaling resistance against NaCl in each treatment group, which was defined as follows:^[^ ^55^ ^]^

(6) $$Approximate\;anti\;scaling\;evaluation=\frac{J_{x}}{J_{3.5}}$$

where *J_x_* (LMH °C^−1^) is the flux for the desalination of NaCl solutions at specific concentrations (3.5 wt%, 9.5 wt%, 14 wt%, and 17.5 wt%), and *J_3.5_* (LMH °C^−1^) is the flux for the desalination of a 3.5 wt% NaCl solution.

The scaling resistance against gypsum was evaluated by calculating the normalized flux under different VCFs (volume concentration factors). The VCF and normalized flux were calculated according to the following two equations:

(7) $$VCF=\frac{V_{initial}}{V_{\mathrm{current}}}$$

(8) $$Normalized\;flux=\frac{J_{initial}}{J_{\mathrm{current}}}$$

where *J_initial_* (LMH °C^−1^) and *J_current_* (LMH °C^−1^) are the initial and current fluxes, respectively. *V_initial_* (1000 mL) and *V_current_* (mL) are the initial and current volumes of the CaSO_4_ solution, respectively.

Additionally, the operating conditions for long‐term experiments were consistent with the antifouling assessment procedure. A 10% NaCl solution and a 5 g L^−1^ CaSO_4_ solution were used as the feed solutions, with deionized water and H_2_O_2_ quantitatively replenished every 4 h to maintain a constant concentration throughout the experimental period.​

## Conflict of Interest

The authors declare no conflict of interest.

## Author Contributions

Z.S.L. and Q.C. conceived the research project. Z.S.L. and Q.C. designed and conducted the experiments. Z.S.L. and X.C. conducted the material characterization and analyzed the measurement results. Z.S.L. wrote the manuscript. F.H.A., X.X.W. and Q.C. reviewed and revised the manuscript. All the authors discussed the data and commented on the paper.

## Supporting information



Supporting Information

Supplemental Video 1

Supplemental Video 2

## Data Availability

The data that support the findings of this study are available from the corresponding author upon reasonable request.
