# Supplementary material for: Simultaneously Boosting Freshwater Productivity and Antifouling Efficacy of Membrane Distillation Through In Situ Micro‐Bubble Generation
Source: Adv Sci (Weinh). 2025 Sep 29;12(44):e07246. doi: 10.1002/advs.202507246 (PMC12667539; doi:10.1002/advs.202507246)
Supplement: Supplementary file 1 — Supporting Information [file ADVS-12-e07246-s001.docx]

# Supporting information

**Simultaneously Boosting Freshwater Productivity and Antifouling Efficacy of Membrane Distillation through In-Situ Micro-bubble Generation**

Zhongsheng Li^1,2^, Faheem Hassan Akhtar^3^, Xin Cui^4^, Xiaoxiong Wang^1,2^, Qian Chen^1,2,*^

1. *Shenzhen Key Laboratory of Advanced Technology for Marine Ecology, Tsinghua University, Shenzhen 518055, China*
2. *Institute for Ocean Engineering, Tsinghua Shenzhen International Graduate School, Tsinghua University, Shenzhen 518055, China*
3. *Department of Chemistry and Chemical Engineering, Lahore University of Management Sciences (LUMS), Lahore 54792, Pakistan*
4. *Institute of Building Environment and Sustainable Technology, Xi’an Jiaotong University, Xi’an 710049, China*

*Corresponding author, email: [qian.chen@sz.tsinghua.edu.cn](mailto:qian.chen@sz.tsinghua.edu.cn)

**This file includes:**

Section S1. The experimental equipment setup.

Section S2. The procedure for determining the membrane fabrication parameters.

Section S3. The experimental results of membrane performance.

Section S4. Crystallographic and chemical structures of the membranes

Section S5. Liquid entry pressure (LEP) measurement and results comparison.

Section S6. Heat and mass transfer theories.

Section S7. Mass transfer coefficient.

Section S8. Concentration polarization on the membrane surface.

Section S9. The procedure for developing new Nusselt correlation.

Section S10. The experiments setup for modifying model.

Section S11. The model verification.

Section S12. Characterization of gypsum fouling on the membrane surface.

Section S13. The assessment of membrane intrinsic stability.

Section S14. Nucleation barrier theory.

## Section S15. The procedure for recording in-situ micro-bubbles.

Section S16. The comparison between this work and previous studies.

Section S17. The analysis of economic prospects.

Section S18. The mechanism of catalytic H_2_O_2_ decomposition by MnO_2_.

Figure S1. Schematic of a typical DCMD system.

Figure S2. Physical image of the operating equipment.

Figure S3. Membrane performance in five aspects. Flux, flux with H_2_O_2_, hydrophobicity (water contact angle) and resistance to low surface tension liquid (glycol aqueous contact angle) under different proportions of (A1) γ-MnO_2_ and (A2) FDTS. Antiwetting efficiency (durability during desalination of SDS mixed solution) under different proportions of (B1) γ-MnO_2_ and (B2) FDTS.

Figure S4. Crystallographic and chemical structures of the PM and the pristine membrane. (A) X-ray diffraction (XRD) patterns. (B) X-ray photoelectron spectroscopy (XPS) survey spectra. Deconvoluted core level spectra of (C) Mn 2p and (D) C 1s.

Figure S5. LEP test setup. (A) Schematic. (B) Physical image of the detection cell.

Figure S6. Schematic of the theoretical analysis and model formulation. (A) A typical countercurrent DCMD process. (B) The abstracted module is divided into N control elements. (C) An arbitrary control element.

Figure S7. Flow chart for obtaining an accurate heat transfer coefficient, salt concentration and modified Nusselt number correlation for the in-situ micro-bubble DCMD process.

Figure S8. Modified correlation of the PM with in-situ micro-bubbles and model verification. (A) The modified Nusselt correlation was obtained from the experimental results. The experimental and model simulated results of (B) the feed side outlet temperature, (C) permeate side outlet temperature, and (D) mass flux.

Figure S9. Comparison of scaling in the treatment groups. Cross-sectional SEM images and energy dispersive X-ray spectroscopy (EDS) elemental maps of (A1, C1) the pristine membrane and (B1, D1) the PM before the experiments. SEM and EDS images of (A2, C2) the pristine membrane, (A3, C3) the pristine membrane with H_2_O_2_, (B2, D2) the PM and (B3, D3) the PM_bub after the experiments.

Figure S10. Detection of surface coating material leaching. (A) Photographs of the modified membrane before experiment. Photographs of (B) the PM and (C) the PM_bub after experiment. (D) Desorption rate during the long-term desalination.

Figure S11. The experimental setup for recording the in-situ micro-bubbles.

Figure S12. The flux under different H_2_O_2_ concentrations

Table S1. The composition details of the prepared parameters.

Table S2. Comparison of the LEP with recently reported modified membranes.

Table S3. The experiments at different *ΔT* with varying flow rates.

Table S4. Comparison of the gypsum solution desalination performance of the PM (this work) with those of various previous studies.

Table S5. Unit MD module cost of pilot-scale systems.

Table S6. Unit Production Cost (*UPC*) of pilot-scale systems (24 m^3^ d).

## **Section S1. The experimental equipment setup.**

The DCMD system consists mainly of a membrane module, two thermostatic water baths, two feed tanks (5 L), two peristaltic pumps, two flow meters, four conductivity detectors and four temperature sensors, as shown in **Figure S1** and **Figure S2**. The channel length, width and height of the membrane module are 49 mm, 40 mm and 2 mm, respectively.


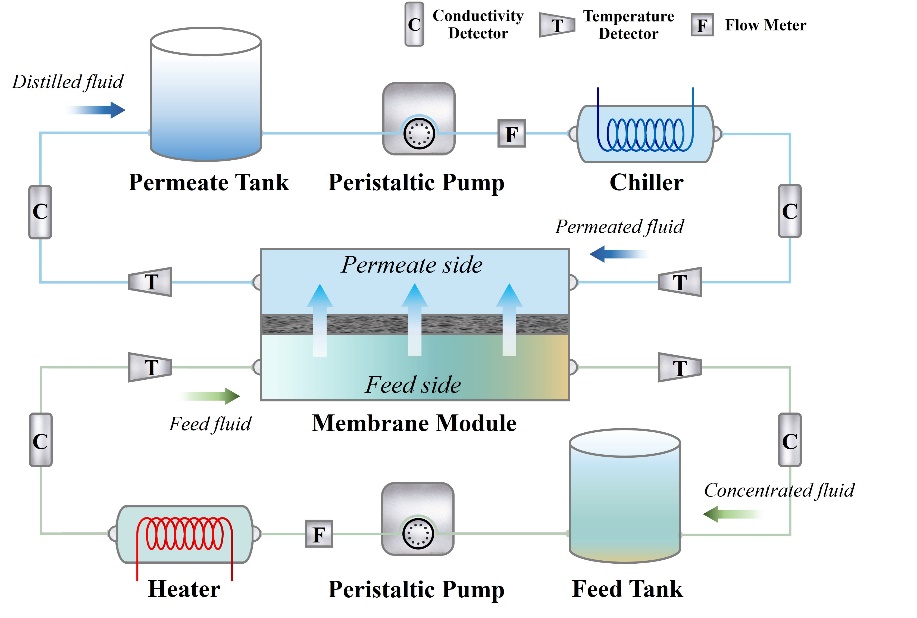


**Figure S1. Schematic of a typical DCMD system.**


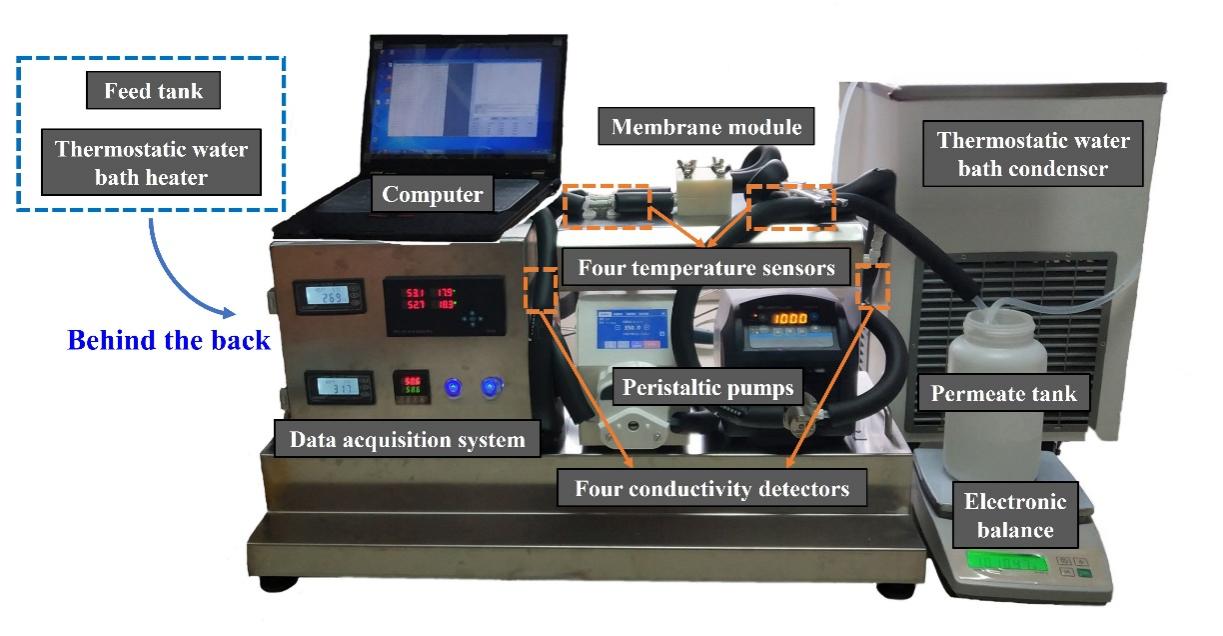


**Figure S2. Physical image of the operating equipment.**

## **Section S2. The procedure for determining the membrane fabrication parameters.**

Eight different modified membranes were prepared to determine the optimal fabrication parameters. Compositions of each membrane are summarized in **Table S1**.

**Table S1.** The composition details of the prepared parameters.

| Membrane | γ-MnO_2_ (g m^-2^) | FDTS (mL m^-2^) |
| --- | --- | --- |
| Modify-1 | 1.132 | 15 |
| Modify-2 | 2.264 | 15 |
| Modify-3 | 4.528 | 15 |
| Modify-4 | 6.792 | 15 |
| Modify-5 | 2.264 | 5 |
| Modify-6 | 2.264 | 10 |
| Modify-7 | 2.264 | 20 |
| Modify-8 | 2.264 | 30 |

The membrane performance is evaluated based on the five performance variables: flux, flux with H_2_O_2_ (representing the flux with micro-bubble enhancement), hydrophobicity (water contact angle), resistance to low surface tension liquid (glycol aqueous contact angle), and antiwetting efficiency (durability during desalination of SDS mixed solution). The flux of the membranes was measured for 2 hour (7200 s) using 3.5 wt% NaCl solution as the feed. Then 0.1 wt% H_2_O_2_ was added into the feed fluid to measure, and the resulting change in flux was recorded. To facilitate the determination of antiwetting properties, a solution containing sodium dodecyl sulfate (SDS, 0.4 mM) was added to the feed solution (NaCl, 3.5 wt%) to reduce surface tension and accelerate pore wetting. Moreover, a normalized method for comparing membrane properties was established, the antiwetting efficiency of the pristine membrane was normalized to 0.125 (wetting after 900 s of desalination (900 s / 7200 s)), while the other variables were set to 1. These experiments were conducted at a temperature difference (*ΔT = T_Feed_ - T_Permeat_*) of 40℃ with a flow rate of 500 mL min^-1^ on both sides.

The flux was evaluated by the following equation:

$J_{g}=\frac{\Delta m}{\Delta t\cdot\Delta T\cdot A}$ (S1)

where *J_g_* (LMH ℃^-1^) is the flux of the treatment groups, *Δm* (kg) is the mass of the product water, *ΔT* (~2 h) is the measurement time required to reach stability, *ΔT* (℃) is the temperature difference across the membrane, and *A* (~0.001960 m^2^) is the membrane area.

The antiwetting efficiency was defined as follows:

$Antiwetting efficiency=\frac{t_{wetting}}{t_{limited}}$ (S2)

where *t_wetting_* (s) is the time required for pore wetting to occur and *t_limited_* (~7200 s) is the duration of the experiment.

## **Section S3. The experimental results of membrane performance.**

In the fabrication optimization experiments, we evaluate the performance of the membranes using five performance variables as described in **Section S3.** As shown in **Figure S3A1 & A2**, the modified membranes exhibit significantly higher contact angles for both water and glycol aqueous, exceeding 170° and 150°, compared to the pristine membrane. Furthermore, the fluxes initially increase with the rising γ-MnO_2_ and FDTS content but subsequently decrease, achieving the peak values for Mod-2 and Mod-6, respectively. During a 2-hour desalination process with SDS mixed solution (**Figure S3B1 & B2**), all modified membranes remained non-wetted, except for Mod-4, Mod-5, and Mod-6. As a result, Mod-2 exhibits the optimal overall performance and is denoted as the PM.


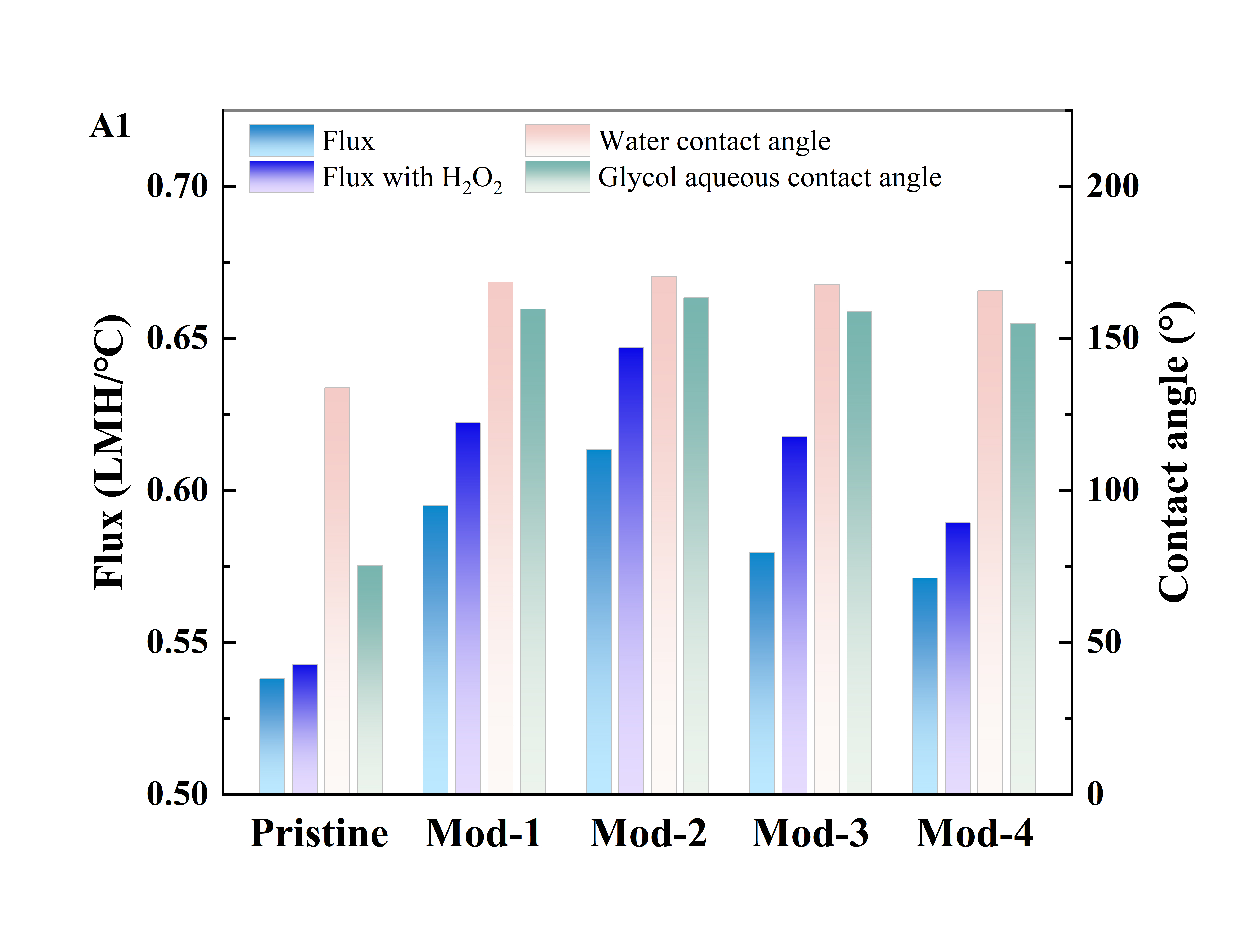

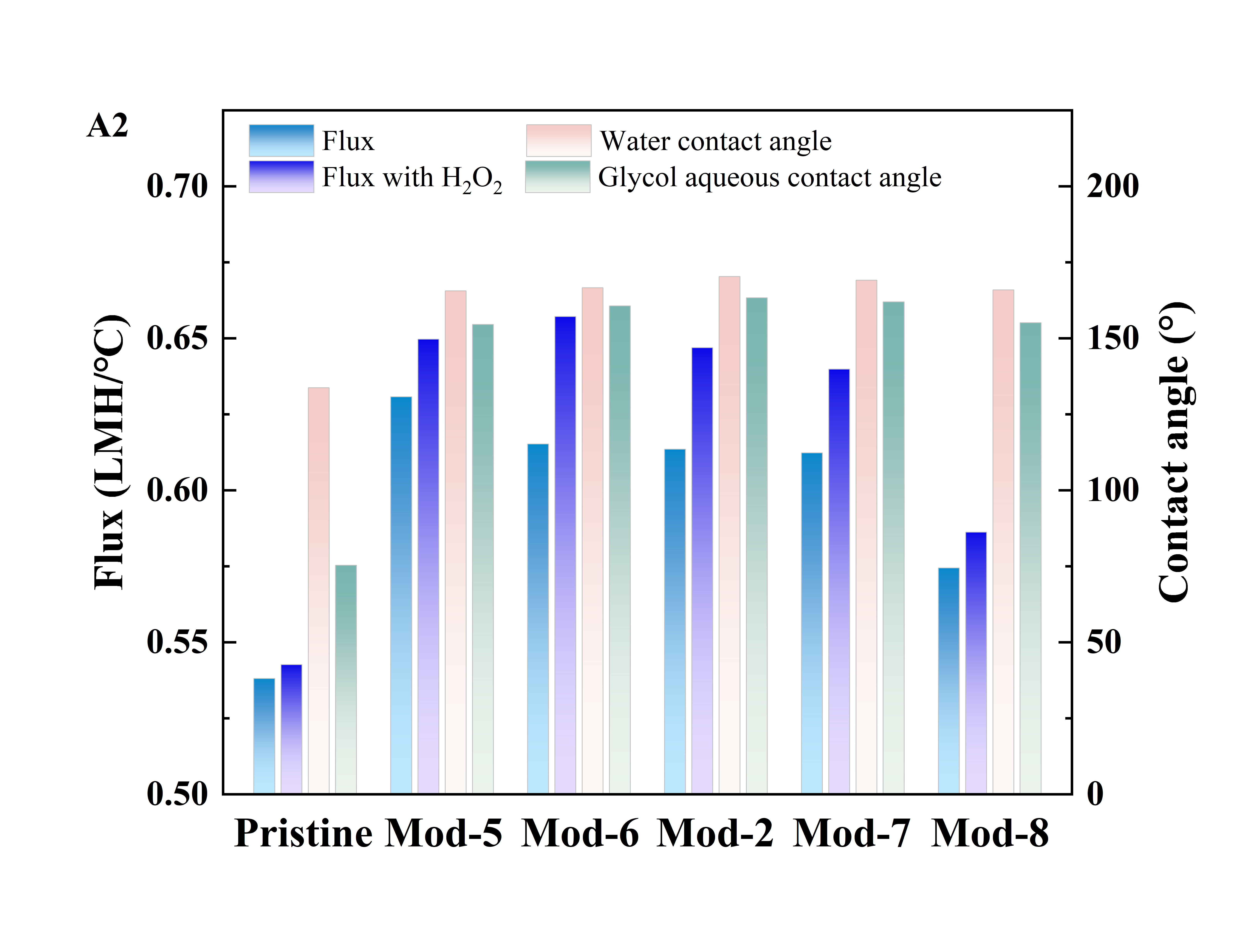

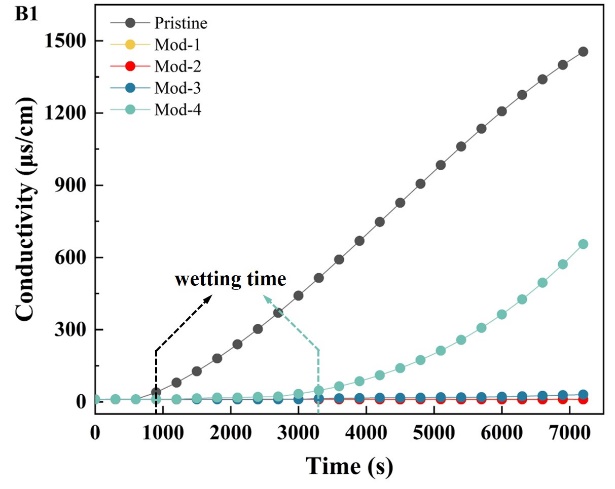

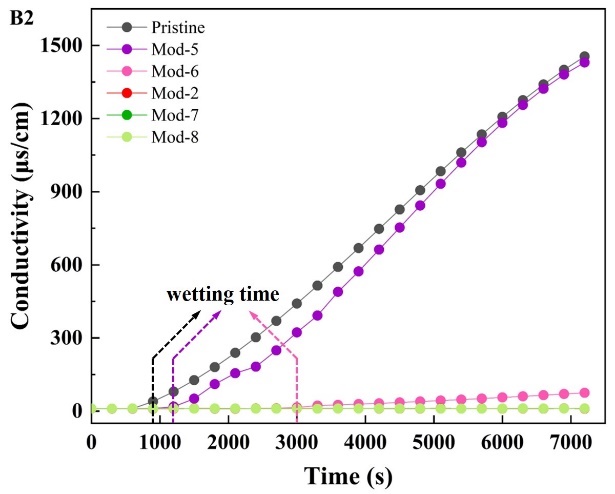


**Figure S3.** **Membrane performance in five aspects.** Flux, flux with H_2_O_2_, hydrophobicity (water contact angle) and resistance to low surface tension liquid (glycol aqueous contact angle) under different proportions of **(A1)** γ-MnO_2_ and **(A2)** FDTS. Antiwetting efficiency (durability during desalination of SDS mixed solution) under different proportions of **(B1)** γ-MnO_2_ and **(B2)** FDTS.

## **Section S4. Crystallographic and chemical structures of membranes.**

As shown in **Figure S4A**, the high purity γ-MnO_2_ utilized in the experiments closely resembling the γ-MnO_2_ standard (JCPDS No. 014-0644). The similar peaks observed in both the pristine membrane and the PM correspond to the crystal planes of polyvinylidene difluoride (PVDF)^[1]^, and the extra diffraction peaks exhibited in the PM are attributed to the γ-MnO_2_ crystal planes^[2]^. Remarkably, the peak intensity of the PM is weaker than that of the pristine membrane, indicating the possible influences of the particle size distribution and surface roughness due to the addition of FDTS^[3]^. On the other hand, the XPS survey spectrum of the PM (**Figure S4B**) reveals new peaks corresponding to Mn, O, and Si. In the Mn (2p) region (**Figure S4C**), two prominent spectral features are observed in the PM and γ-MnO_2_ nanofilaments, suggesting that the mixture attached to the membrane effectively inherited the same crystal structure and properties as the pure chemical^[4]^. As presented in the C (1s) spectrum (**Figure S4D**), in addition to the components (CF_2_, CH_2_ and C-H) associated with the main chains of PVDF, there are three new peaks at binding energies of 292.9 eV (CF_3_), 288.1 eV (CH_2_-CF_2_) and 286.2 eV (CH_2_-CF_2_) for the PM, which are attributed to FDTS^[5]^. On the basis of the above analysis, a mixture of γ-MnO_2_ and FDTS is successfully coated on the PM.

**
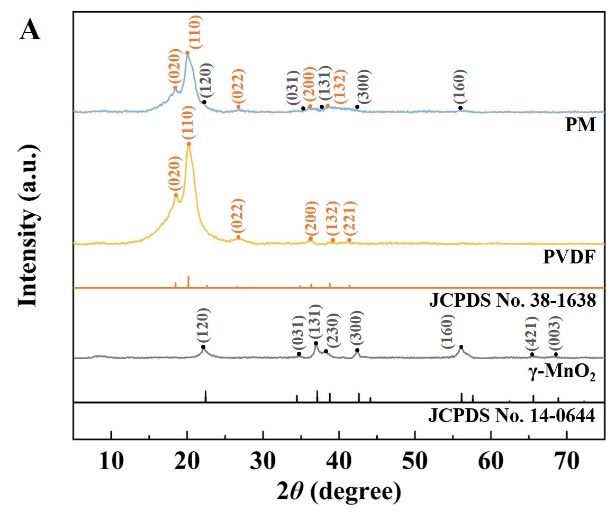

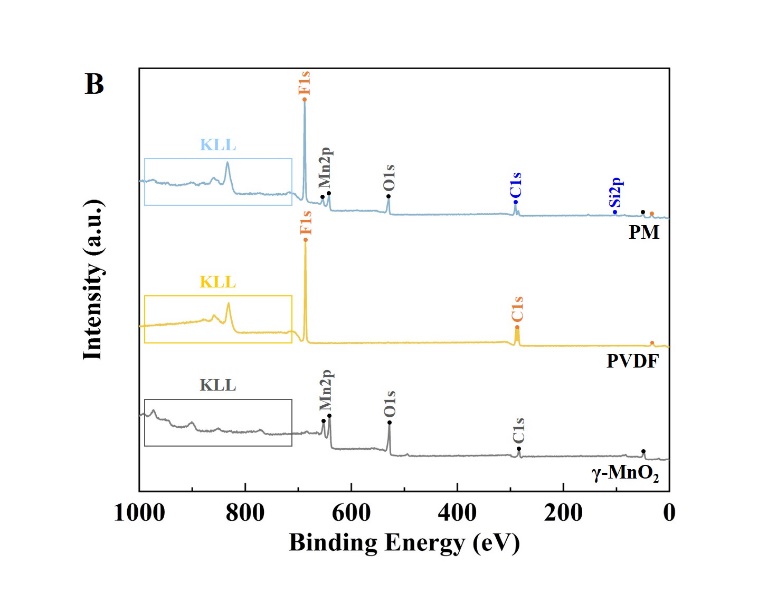

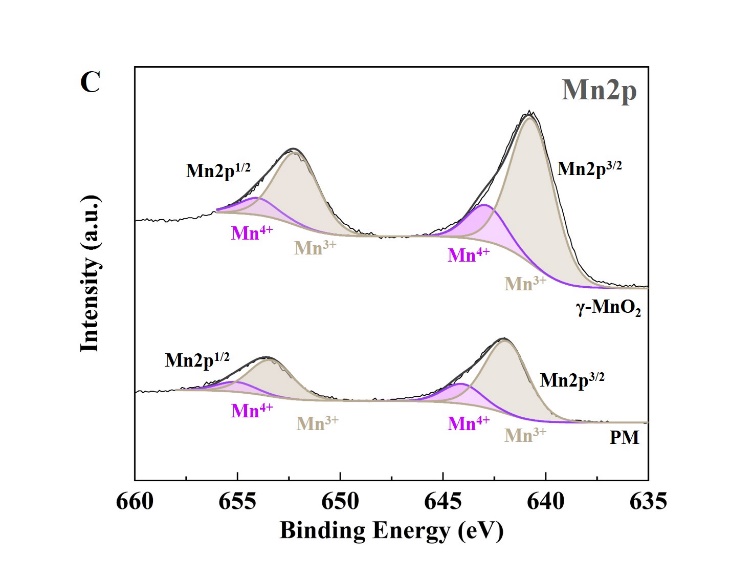

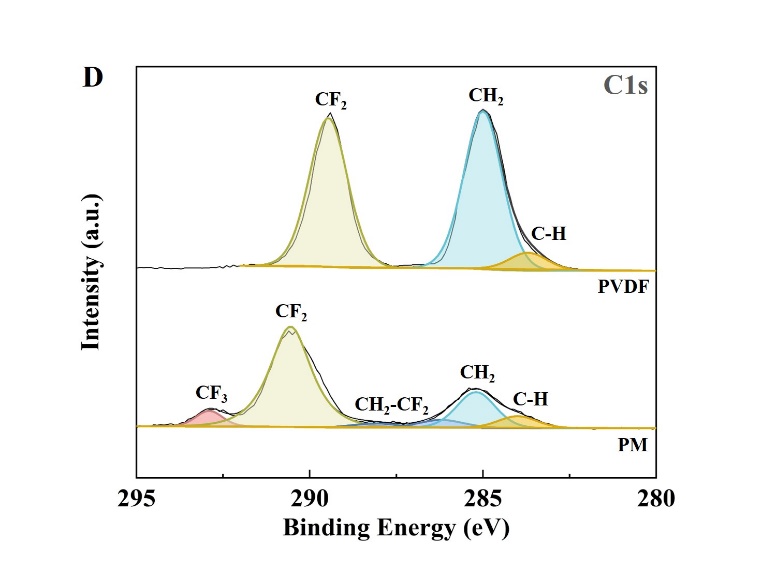
**

**Figure S4. Crystallographic and chemical structures of the PM and the pristine membrane. (A)** X-ray diffraction (XRD) patterns. **(B)** X-ray photoelectron spectroscopy (XPS) survey spectra**.** Deconvoluted core level spectra of **(C)** Mn 2p and **(D)** C 1s.

## **Section S5. Liquid entry pressure (LEP) measurement and results comparison.**

Liquid entry pressure (LEP) is defined as the minimum transmembrane pressure required for liquid to penetrate through the membrane pore. In this work, LEP measurements were conducted using a detection cell, where deionized water was pressurized with gas at a gradual increment of 50 kPa (**Figure S5**). Once water permeation occurred (the first droplet spilled out from the cell), the corresponding pressure was recorded as the LEP. According to the test results, the LEP of the pristine membrane and the PM (the modified membrane of this work) are 145 kPa and 375 kPa, respectively. As shown in Table S2, compared with the recently reported PVDF flat-sheet modified membranes, the PM exhibits a significantly higher LEP and a notable increased ratio.


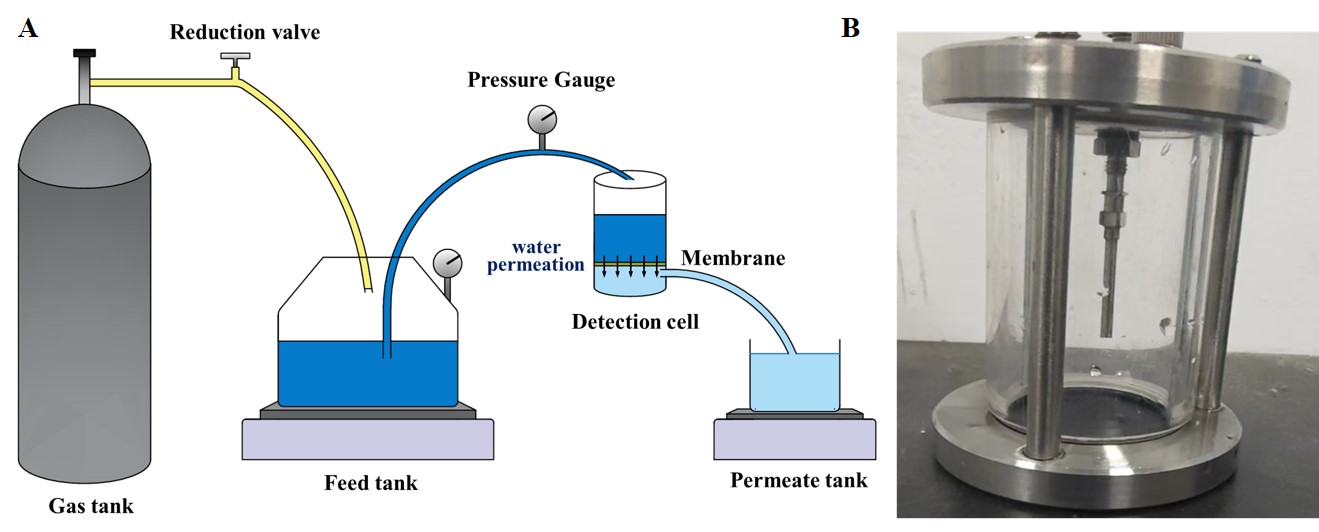


**Figure S5. LEP test setup. (A)** Schematic. **(B)** Physical image of the detection cell.

**Table S2.** Comparison of the LEP with recently reported modified membranes.

| References | Modified method | Pristine pore size (μm) | Pristine LEP (kPa) | Modified LEP (kPa) | Increased ratio (%) |
| --- | --- | --- | --- | --- | --- |
| This work | Commercial membrane modified | 0.45 | 145 | 375 | 159 |
| [6] |  | 0.22 | 260 | 360 | 38 |
| [7] |  | 0.22 | 130 | 210 | 62 |
| [8] |  | 0.22 | 100 | 290 | 190 |
| [9] | Electrospinning membrane modified | 0.45 | 130 | 160 | 23 |
| [10] |  | 0.22 | 120 | 300 | 150 |
| [11] | Phase inversion membrane modified | 0.45 | 210 | 280 | 33 |
| [12] |  | 0.45 | 220 | 280 | 27 |

**Section S6. Heat and mass transfer theories.**

The DCMD process involves simultaneous heat and mass transfer, driven by the temperature difference and vapor pressure gradient across the membrane, as shown in **Figure S6A.** The module is abstracted and divided into N control elements along the flow direction (**Figure S6B**). Within an arbitrary control element (**Figure S6C**), the heat transfer from the feed side fluid to the membrane surface is provided by the following equation:

$Q_{Fi}=h_{Fi}\cdot(T_{Fi}-T_{Fmi})\cdot dA$ (S3)


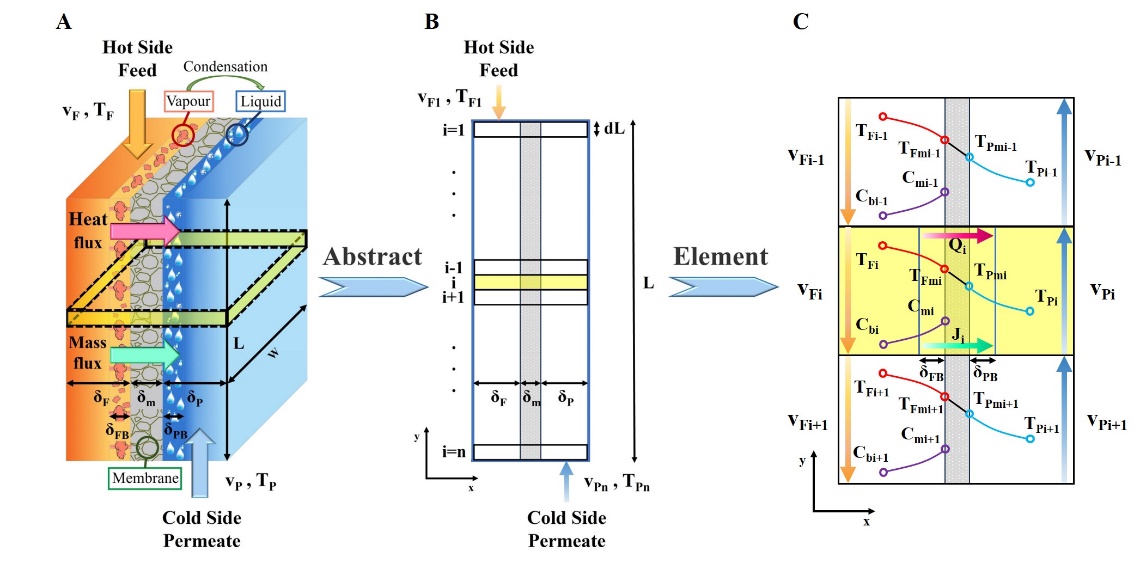
where *h_Fi_* (W m^-2^·K^-1^) and *T_Fi_* (K) are the heat transfer coefficient and temperature of the feed side fluid, respectively; *T_Fmi_* (K) is the membrane surface temperature of the feed side, and *dA* (m^2^) is the membrane area.

**Figure S6. Schematic of the theoretical analysis and model formulation.** **(A)** A typical countercurrent DCMD process. **(B)** The abstracted module is divided into N control elements. **(C)** An arbitrary control element.

The heat transfer coefficient is estimated via the following correlation:

 (S4)

where *Nu_i_* represents the Nusselt number, *λ_i_* (W m^-1^·K^-1^) represents the thermal conductivity of the fluid, and *D_i_* (m) represents the hydraulic diameter.

Similarly, the heat transfer from the membrane surface on the permeate side to the fluid is given as follows:

$Q_{Pi}=h_{Pi}\cdot(T_{Pmi}-T_{Pi})\cdot dA$ (S5)

where *h_Pi_* (W m^-2^·K^-1^) and *T_Pi_* (K) are the heat transfer coefficient and temperature of the permeate side fluid, respectively, and *T_Pmi_* (K) is the membrane surface temperature of the permeate side.

Specifically, the heat transferred across the membrane, including the latent heat of vapor transition and heat conduction through the membrane, is expressed as:

$Q_{mi}=[J_{i}\cdot h_{fg}^{*}-\frac{\lambda_{m}}{\delta_{m}}\cdot(T_{Pmi}-T_{Fmi})]\cdot dA$ (S6)

where *J_i_* (kg m^-2^·s^-1^) is the mass flux, *h_fg_^*^* (J kg^-1^) is the enthalpy of vaporization, *λ_m_* (W m^-1^·K^-1^) represents the thermal conductivity of the membrane, and *δ_m_* (m) is the membrane thickness.

The mass flux *(J)* is assumed to be proportional to the vapor pressure difference across the membrane, and is calculated via the following equation^[13]^:

$J_{i}=B_{i}\cdot(P_{TF,mi}-P_{TP,mi})$ (S7)

where *B_i_* (kg m^-2^·Pa^-1^·s^-1^) represents the membrane mass transfer coefficient, and the caculation method is provided in **Section S7**. *P_TF,mi_* (Pa*)* and *P_TP,mi_* (Pa) are the vapor pressures on both sides of the membrane, which are correlated with the salt concentration and the temperature on the membrane surface, as defined as follows^[14]^:

$P_{m}=f(T_{m},C_{m})$ (S8)

where *P_m_* (Pa), *T_m_* (K), and *C_m_* (wt%) are the saturation (vapor) pressure, temperature and salt concentration on the membrane surface, respectively.

The concentration polarization arises from the phenomena of vapor permeation and salt accumulation on the membrane surface, and is defined as follows^[15]^:

$Concentration polarization coefficient (CPC)=\frac{C_{mi}}{C_{bi}}$ (S9)

where *C_bi_* (wt%) is the salt concentration in the bulk solution, and *C_mi_* (wt%) is the salt concentration on the membrane surface. Their estimation method is provided later in the **Section S8**.

Under steady-state conditions, the amounts of heat transfer on both sides of the membrane and across the membrane are equal:

$Q_{Fi}=Q_{mi}=Q_{Pi}$ (S10)

As shown in **Figure S6C**, mass and energy conservation relationships also exist between two consecutive control elements^[16]^. The mass conservation is expressed as:

$v_{Fi+1}=v_{Fi}- J_{i}\cdot dA$ (S11)

$v_{Pi+1}=v_{Pi}- J_{i}\cdot dA$ (S12)

where *v_Fi_* (kg s^-1^) and *v_Pi_* (kg s^-1^) are the mass flow rates of the feed side fluid and permeate side fluid, respectively.

Considering minimal changes in the mass flow rate and specific heat between adjacent control elements, the energy conservation relation is defined as follows:

$Q_{Fi}=(T_{Fi}-T_{Fi+1}) \cdot v_{Fi}\cdot c_{Fi}$ (S13)

$Q_{Pi}=(T_{Pi}-T_{Pi+1}) \cdot v_{Pi}\cdot c_{Pi}$ (S14)

where *c_Fi_* (J kg^-1^·K^-1^) and *c_Pi_* (J kg^-1^·K^-1^) are the specific heats of the feed side fluid and permeate side fluid, respectively.

## **Section S7. Mass transfer coefficient.**

The vapor diffusion inside the membrane pores is a combination of Knudsen diffusion and molecular diffusion; thus, the mass transfer coefficient (*B*) can be estimated via the following four equations^[17]^:

$B=[\frac{P_{\text{air}}\cdot\tau_{\text{m}}\cdot\delta_{m}\cdot T_{m}\cdot R}{P\cdot k\cdot\varepsilon_{\text{m}}\cdot M}+\frac{3\tau_{\text{m}}\cdot\delta_{m}}{2r_{p}\cdot\varepsilon_{\text{m}}}(\frac{\pi T_{m}\cdot R}{8M})^{0.5}]^{-1}$ (S15)

 (S16)

$P_{air}=P-P_{T_{m}}$ (S17)

$P\cdot k=1.895 \text{×}10^{-5}T_{m}^{2.072}$ (S18)

where *τ_m_, T_m_* (K)*, R* (~8.314 J mol^-1^·K^-1^)*, ε_m_, M* (kg mol^-1^) and *r_p_* (m) represent the tortuosity, average membrane temperature, gas constant, porosity, molecular weight of water and average pore size, respectively. *P_air_* (Pa) is the air pressure in the membrane pores, *P* (Pa) is the total pressure in the membrane pores and is assumed to be at atmospheric pressure (~101325 Pa), *P_Tm_* (Pa) is the average vapor pressure in the membrane pores, and *k* (m^2^ s^-1^) represents the water diffusion coefficient.

Since the thin coating has a negligible effect on the thermal conductivity, all the membranes in this work are assumed to be composed of homogeneous PVDF material. As a result, the thermal conductivity of a membrane is defined as follows^[18]^:

$\lambda_{m}=\varepsilon_{\text{m}}\cdot\lambda_{\text{v}}+(1-\varepsilon_{\text{m}})\cdot\lambda_{s}$ (S19)

$\lambda_{v}=5.71 \times10^{-5}T_{m}+2.72 \times10^{-3}$ (S20)

$\lambda_{s}=5.77 \text{×}10^{-4}T_{m}+9.23 \times10^{-3}$ (S21)

where *λ_v_* (W m^-1^·K^-1^) represents the vapor thermal conductivity through the membrane pores and *λ_s_* (W m^-1^·K^-1^) represents the thermal conductivity of the PVDF material.

## **Section S8. Concentration polarization on the membrane surface.**

As shown in **Figure S6C**, there is a difference in salt concentrations between the bulk and the membrane surface on the feed side. The concentration on the membrane surface (*C_m_*) is estimated via the following three equations^[19]^:

$J=\rho\cdot\frac{k\cdot Sh}{D}\cdot ln\frac{C_{m}}{C_{b}}$ (S22)

$Sh=0.13Re^{0.64}\cdot Sc^{0.38}$ (S23)

$Sc=\frac{\mu}{\rho\cdot k}$ (S24)

where *C_m_* (wt%) and *C_b_* (wt%) are the salt concentrations on the membrane surface and in the bulk solution, respectively; *J* (kg m^-2^·s^-1^) is the mass flux, *ρ* (kg m^-3^) represents the density, *μ* (kg m^-1^·s^-1^) represents the dynamic viscosity, *k* (m^2^ s^-1^) represents the water diffusion coefficient, *D* (m) represents the hydraulic diameter, *Sh* represents the Sherwood number, and *Sc* represents the Schmidt number.

## **Section S9. The procedure for developing new Nusselt correlation.**

The experimental results under various operating conditions (**Section S10**), including the mass flux (J), the inlet and outlet temperatures of the feed side (*T_Fin_ & T_Fout_*), and the inlet and outlet temperatures of the permeate side (*T_Pin_ & T_Pout_*), are incorporated into the model to obtain the equivalent heat transfer coefficient (*h_F_*) and *CPC* for the DCMD process, and the calculation procedure is illustrated in **Figure S7.** First, the basic physical properties of the module and membrane are imported. An initial *h’_F_* and *C_m_* (salt concentration on the membrane surface) are then assumed, and the corresponding *T’_Fout_, T’_Pout_* and *J’* values are subsequently calculated. Finally, the values of *h’_F_* and *C_m_* are modified in the main loop until the calculated temperatures and flux simultaneously match the experimental data.

Finally, the corresponding Reynolds (*Re*), Prandtl (*Pr*), and Nusselt (*Nu*) numbers of the PM with in-situ micro-bubbles are used to modify the Nusselt number correlation for the novel DCMD process via the following format^[20]^:

$Nu=a\cdot Re^{b}\cdot Pr^{c}$ (S25)

where *a*, *b* and *c* are the coefficients.


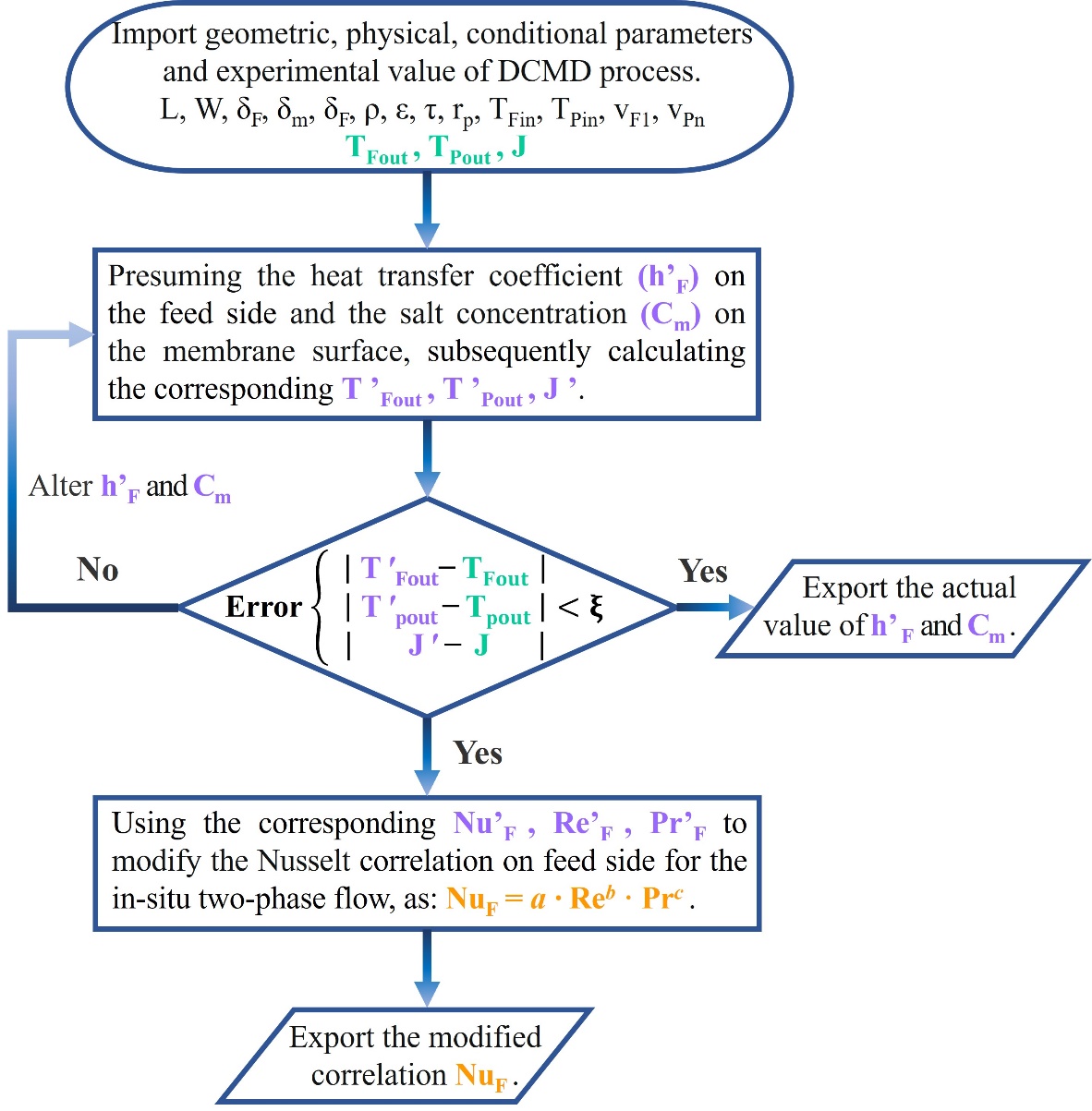


**Figure S7. Flow chart for obtaining an accurate heat transfer coefficient, salt concentration and modified Nusselt number correlation for the in-situ micro-bubble DCMD process.**

## **Section S10. The experiments setup for modifying model.**

Forty-six sets of experiments were performed to investigate the performance of the treatment groups and obtain modified heat transfer correlation for the in-situ micro-bubble DCMD process.

**Table S3.** The experiments at different *ΔT* with varying flow rates.

| Treatment group | No. | Flow rate (mL min^-1^) | *ΔT* (℃) |
| --- | --- | --- | --- |
| PM without H_2_O_2_ | 1 | 250 | 20 |
|  | 2 | 500 |  |
|  | 3 | 750 |  |
|  | 4 | 1000 |  |
|  | 5 | 250 | 30 |
|  | 6 | 500 |  |
|  | 7 | 750 |  |
|  | 8 | 1000 |  |
|  | 9 | 250 | 40 |
|  | 10 | 500 |  |
|  | 11 | 750 |  |
|  | 12 | 1000 |  |
|  | 13 | 250 | 50 |
|  | 14 | 500 |  |
|  | 15 | 750 |  |
|  | 16 | 1000 |  |
| PM with H_2_O_2_ | 17 | 250 | 20 |
|  | 18 | 500 |  |
|  | 19 | 750 |  |
|  | 20 | 1000 |  |
|  | 21 | 250 | 30 |
|  | 22 | 500 |  |
|  | 23 | 750 |  |
|  | 24 | 1000 |  |
|  | 25 | 250 | 40 |
|  | 26 | 500 |  |
|  | 27 | 750 |  |
|  | 28 | 1000 |  |
|  | 29 | 250 | 50 |
|  | 30 | 500 |  |
|  | 31 | 750 |  |
|  | 32 | 1000 |  |

| Treatment group | No. | Flow rate (mL min^-1^) | *ΔT* (℃) |
| --- | --- | --- | --- |
| The pristine membrane without H_2_O_2_ | 33 | 250 | 40 |
|  | 34 | 500 |  |
|  | 35 | 750 |  |
|  | 36 | 1000 |  |
|  | 37 | 500 | 20 |
|  | 38 |  | 30 |
|  | 39 |  | 50 |
| The pristine membrane  with H_2_O_2_ | 40 | 250 | 40 |
|  | 41 | 500 |  |
|  | 42 | 750 |  |
|  | 43 | 1000 |  |
|  | 44 | 500 | 20 |
|  | 45 |  | 30 |
|  | 46 |  | 50 |

## **Section S11. The model verification.**

The velocity and concentration field on the feed side are changed when micro-bubbles occur, thereby affecting the DCMD performance. As a result, conventional heat transfer correlations are insufficient for accurately predicting experimental results^[21]^. By using the algorithm presented in **Section S9**, the Reynolds number (Re), Prandtl number (*Pr*), and Nusselt number (*Nu*) under various operating conditions were calculated, and a modified Nusselt correlation was obtained with an R^2^ value of 0.96 (**Figure S8A**), as follows:

$Nu=0.0123Re^{1.042}\cdot Pr^{1.029}$ (S26)

The modified correlation significantly enhances the model tracking accuracy for DCMD behavior, as illustrated in **Figure S8B-****S8D**. The model predictions clearly exhibit a trend similar to that of the experimental results, and the average errors of the feed side outlet temperature *(T_Fout_*), permeate side outlet temperature (*T_Pout_*) and mass flux (*J*) are 0.79%, 1.34% and 1.08%, respectively. These discrepancies can be attributed to factors such as the inherent limitations of experimental measurement accuracy and inevitable heat loss^[22]^. In summary, with the implementation of the modified correlation, the current model demonstrates greater accuracy than the existing numerical models^[23, 24]^.


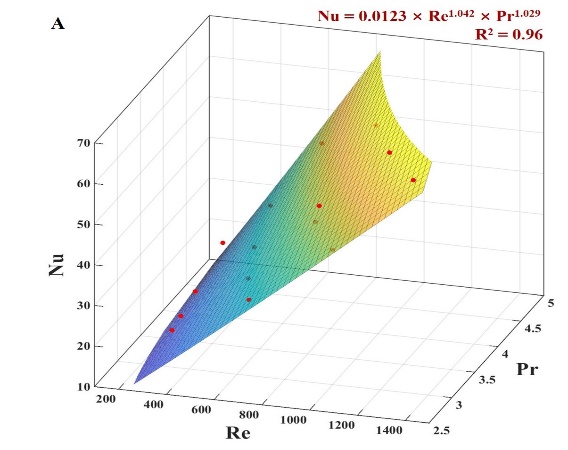

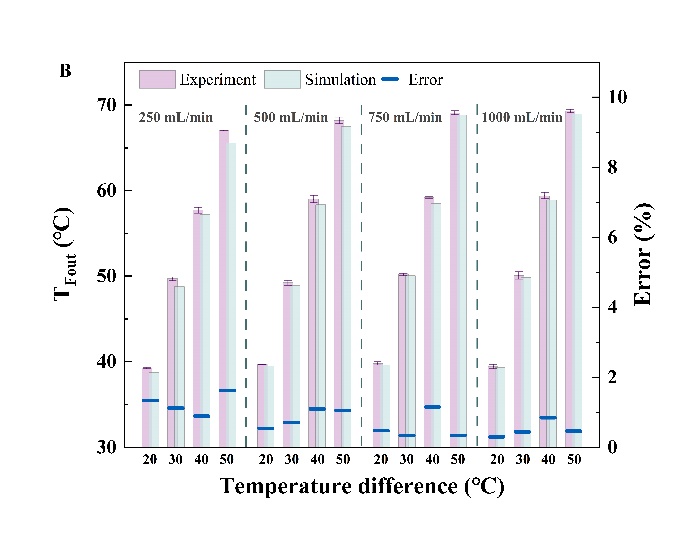

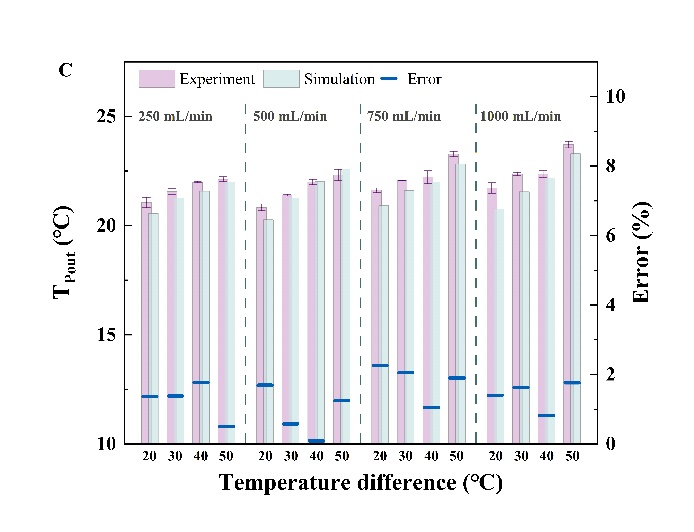

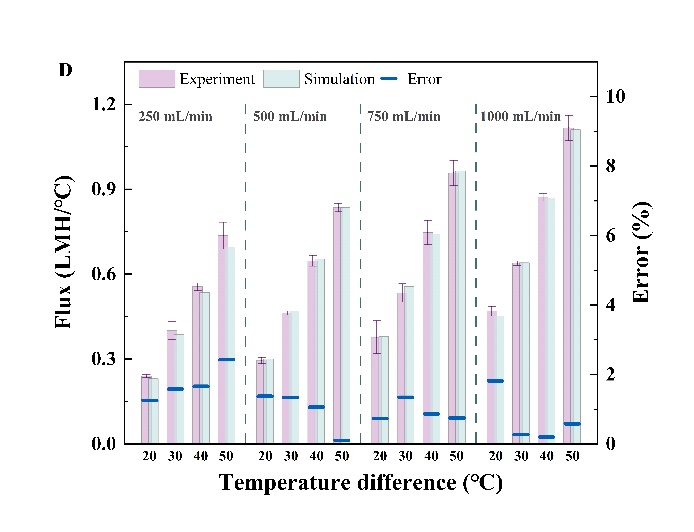


**Figure S8. Modified correlation of the PM with in-situ micro-bubbles and model verification.** **(A)** The modified Nusselt correlation was obtained from the experimental results. The experimental and model simulated results of **(B)** the feed side outlet temperature, **(C)** permeate side outlet temperature, and **(D)** mass flux.

## **Section 12. Characterization of gypsum fouling on the membrane surface.**

Cross-sectional SEM images and energy dispersive X-ray spectroscopy (EDS) maps of the membrane surface clearly reflect the resistance of each treatment group to gypsum. Observations of the untreated pristine membrane and PM are shown in **Figure S9(A1, C1) & S9(B1, D1)**, respectively. After the experiment, heavy "needle-like" scaling is evident on the pristine membrane, with an average thickness of 120 μm (**Figure S9(A2, A3) & S9(C2, C3)**). Furthermore, some thicker areas appear on the pristine membrane surface, where crystal growth causes local structural deformation and induces pore wetting by reducing the liquid entry pressure. In comparison, only a 35 μm scaling layer of gypsum is observed on the PM (**Figure S9(B2, D2)**). Even better, the scaling and Ca detected on the PM_bub surface are almost negligible with the in-situ micro-bubbles (**Figure S9(B3, D3)**).


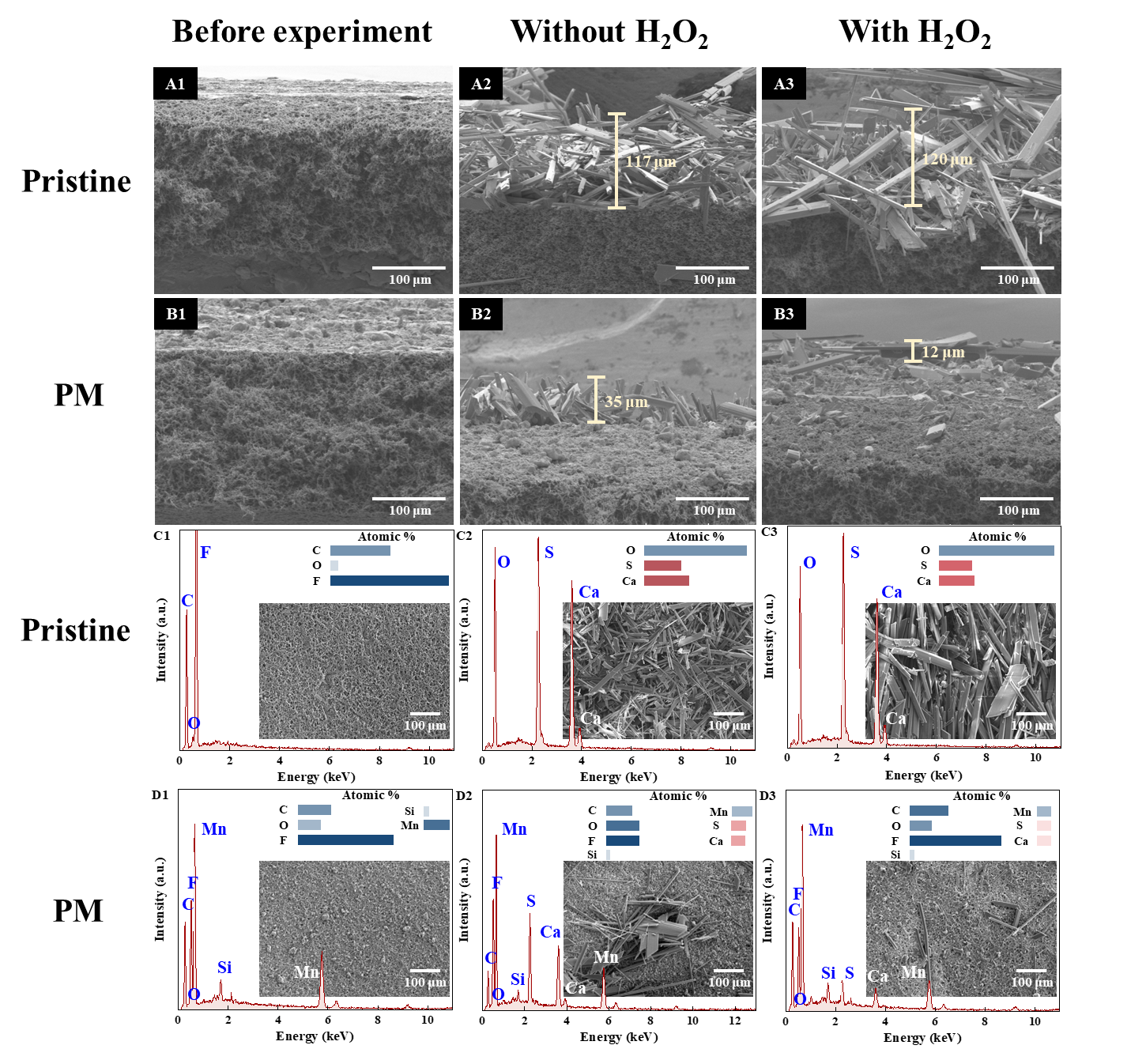


**Figure S9.** **Comparison of scaling in the treatment groups.** Cross-sectional SEM images and energy dispersive X-ray spectroscopy (EDS) elemental maps of **(A1, C1)** the pristine membrane and **(B1, D1)** the PM before the experiments. SEM and EDS images of **(A2,** **C2)** the pristine membrane, **(A3, C3)** the pristine membrane with H_2_O_2_, **(B2, D2)** the PM and **(B3, D3)** the PM_bub after the experiments.

## **Section S13. The assessment of membrane intrinsic stability.**

Compared to the initial state of the modified membranes (**Figure S10A**) before the experiment, material loss from the membrane surface is observed in the PM (**Figure S10B**) and the PM_bub (**Figure S10C**) after long-term DCMD desalination with NaCl solution. To quantify the leaching of surface coating materials, the manganese ion concentration (mg L^-1^) in the feed liquid was detected at 6-hour intervals using inductively coupled plasma (ICP) analysis. As illustrated in **Figure S10D**, the PM and the PM_bub lost 1.5% and 12% of the loaded materials over 60 hours, respectively. Although the PM_bub exhibits greater leaching, about 75% of its total loss occurred within the first 36 hours, after which the desorption rate declines markedly. During the final 6-hour period (54–60 h), the desorption rate of the PM_bub decreased to approximately 0.3%——comparable to that of the PM. These results indicate a critical threshold for the desorption rate, primarily due to the release of inadequately bounded materials on the surface.


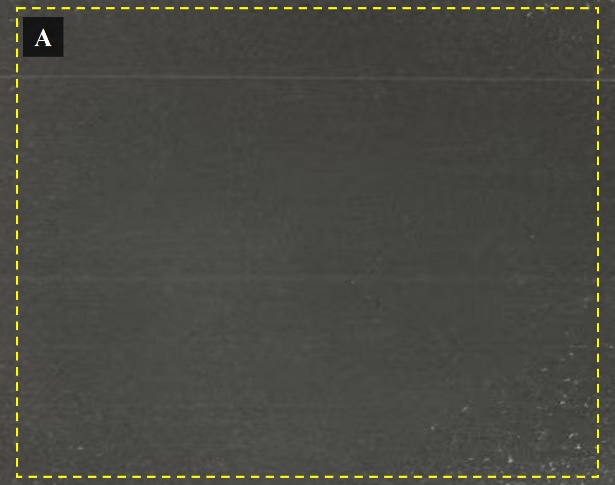

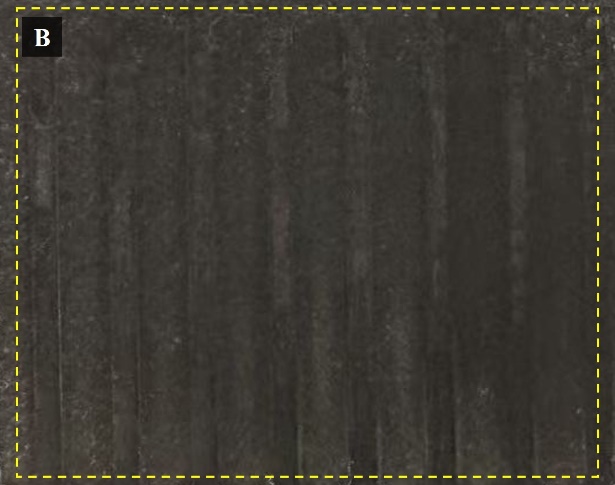

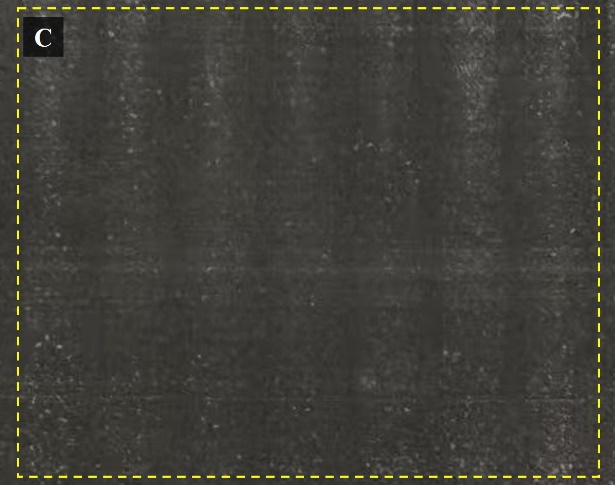

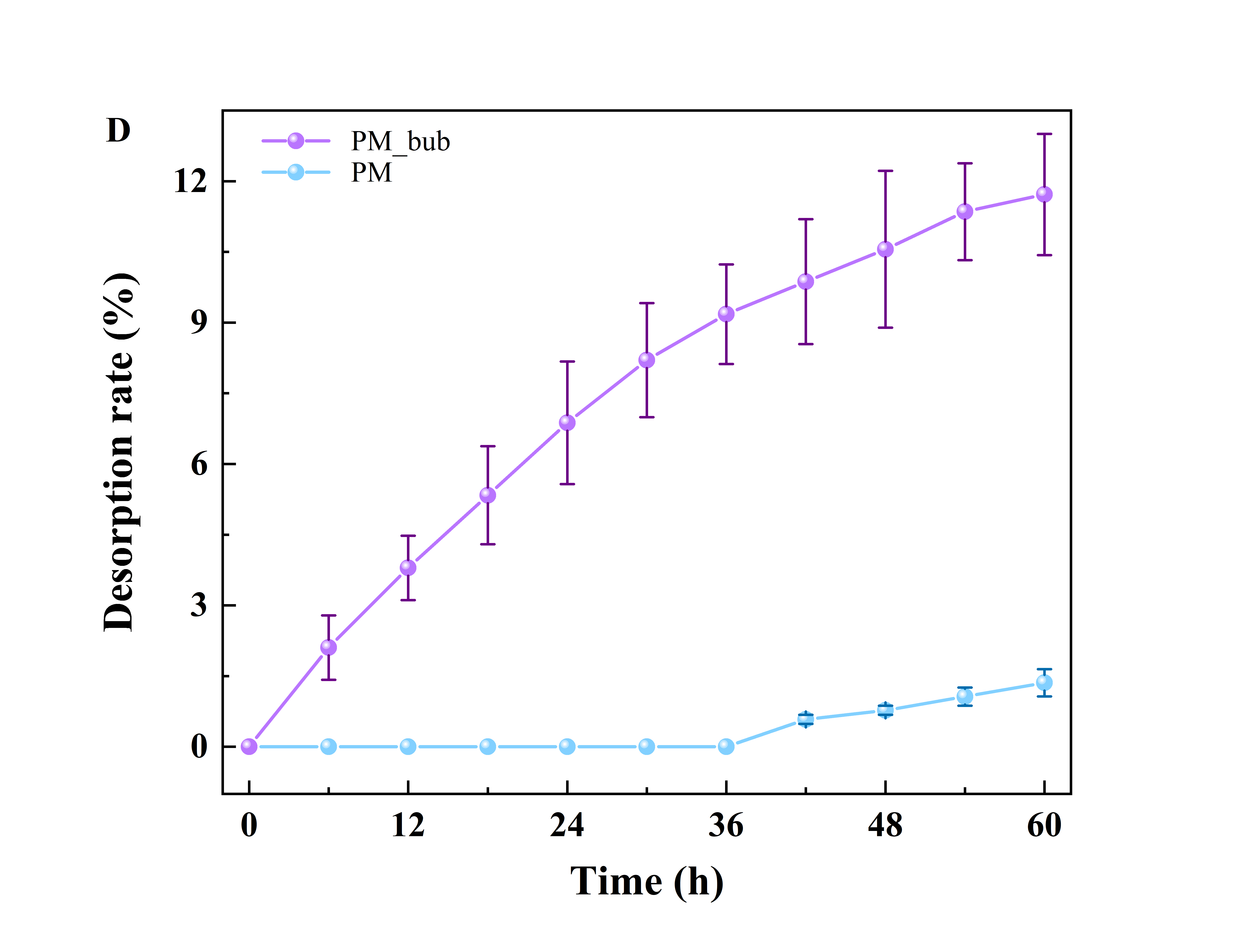


**Figure S10. Detection of surface coating material leaching.** **(A)** Photographs of the modified membrane before experiment. Photographs of **(B)** the PM and **(C)** the PM_bub after experiment. **(D)** Desorption rate during the long-term desalination.

## **Section S14. Nucleation barrier theory.**

The Gibbs free energy barrier is used to quantitatively evaluate the nucleation probability via the following two equations^[25]^:

$\Delta G_{homogeneous}=\frac{\beta\cdot\Gamma^{3}\cdot V^{2}}{(\kappa T)^{2}\cdot ln^{2}S}$ (S27)$\Delta G_{heterogeneous}=\Delta G_{homogeneous}\cdot\left[\frac{1}{4}(2+cos\theta)\cdot(1-cos\theta)^{2}\right]\cdot\left[1-\varepsilon\cdot\frac{(1+cos\theta)^{2}}{(1-cos\theta)^{2}}\right]^{3}$ (S28)

where *ΔG_homogeneous_* and *ΔG_heterogeneous_* represent the Gibbs free energy barriers for homogeneous and heterogeneous nucleation, respectively; *β* represents the surface geometrical factor, *ᴦ* (J m^2^) represents the surface energy of the salt, *V* (m^3^) represents the molecular volume of salt, *κ* (~1.381×10^-23^ J K^-1^) represents the Boltzmann constant, *T* (K) represents the temperature, *S* represents the supersaturation index of the solution, and *θ* (°) represents the contact angle.

## **Section S15. The procedure for recording in-situ micro-bubbles.**

The dynamic behavior of the bubbles in the module was tracked and recorded by a charge-coupled device camera (250 frames), and the experimental setup is depicted in **Figure S11**. A large amount of gypsum was predeposited to enhance the visibility of changes in the scale layer. The initial concentration of H_2_O_2_ was set at 0.1 wt%, as in other experiments. The test lasted 30 seconds under static conditions.


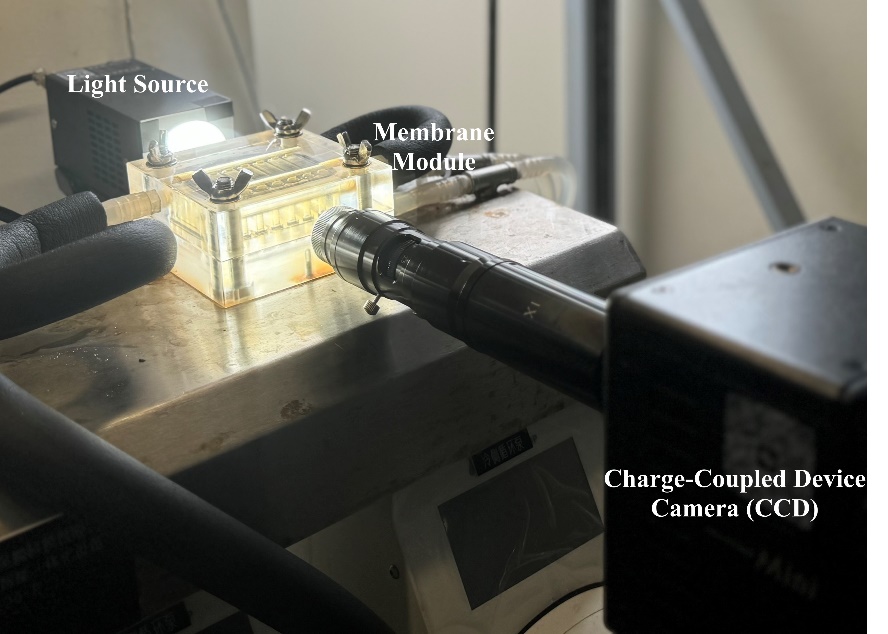


**Figure S11. The experimental setup for recording the in-situ micro-bubbles.**

## **Section S16. The comparison between this work and previous studies.**

In the comparison data, the production enhancement ratio is defined as the ratio of the initial flux for the distilling gypsum solution before and after modification, and the antifouling enhancement ratio is calculated as the ratio of the maximum VCF before and after modification when there is no decline in the normalized flux.

**Table S4.** Comparison of the gypsum solution desalination performance of the PM (this work) with those of various previous studies.

| Reference | Coating method | Modified superhydrophobic  PVDF membrane | Gypsum solution concentration (g L^-1^) | Operating temperature (℃) | Flux before modified (LMH ℃^-1^) | Flux after modified (LMH ℃^-1^) | Enhanced ratio of flux (%) | Antifouling before modified (VCF) | Antifouling after modified (VCF) | Enhanced ratio of  antifouling  (%) |
| --- | --- | --- | --- | --- | --- | --- | --- | --- | --- | --- |
| [41] | Electospraying coating | PVDF-HFP electrospinning + SiNPs with 17-FAS fluorinated. | 2 | 75-20 | 0.731 | 0.809 | 10.70 | 1.17 | 1.35 | 15.38 |
| [45] |  | PVDF electrospinning + SiNPs with 17-FAS fluorinated. | 2.7 | 60-20 | 0.525 | 0.388 | -26.19 | 1.08 | 1.20 | 11.11 |
| [46] |  | F-POSS PVDF-HFP with carbon black. | 2 | 60-20 | 0.563 | 1.000 | 77.78 | 1.25 | 1.43 | 14.40 |
| [47] | Dip  coating  Dip  coating | Commercial membrane + SiNPs with17-FAS fluorinated. | 2.4 | 60-20 | 0.658 | 0.548 | -16.73 | 1.65 | 1.67 | 1.21 |
| [48] |  | Commercial membrane + PVA-GA. | 2.7 | 60-20 | 0.339 | 0.179 | -47.19 | 1.02 | 1.82 | 78.43 |
| [49] |  | Commercial membrane + epoxy acrylic with PFTS fluorinated. | 2 | 60-20 | 0.613 | 0.398 | -35.10 | 1.45 | 2.10 | 44.83 |
| [50] |  | PVDF electrospinning + SiNPs with17-FAS fluorinated. | 2.7 | 60-20 | 0.975 | 0.875 | -10.26 | 1.12 | 1.20 | 7.14 |
| [51] |  | Commercial membrane + TEOS with FTDS fluorinated. | 2 | 60-20 | 0.522 | 0.472 | -9.59 | 1.12 | 1.18 | 5.36 |
| [52] |  | Commercial membrane + SiO_2_ with PDMS fluorinated + feed side aeration. | 2 | 60-20 | 0.423 | 0.548 | 29.59 | 1.33 | 1.76 | 32.33 |
| [52] |  | Commercial membrane + SiO_2_ with PDMS fluorinated. | 2 | 60-20 | 0.423 | 0.400 | -5.33 | 1.33 | 1.68 | 26.32 |
| [53] | Vaccum coating | PVDF phase inversion + fluorinated CNTs. | 2 | 60-20 | 0.463 | 0.575 | 24.32 | 1.11 | 1.33 | 19.82 |
| This work  [PM with in-situ micro-bubbles] |  | Commercial membrane + γ-MnO_2_ with FTDS fluorinated + in-situ micro-bubble. | 5 | 60-20 | 0.477 | 0.672 | 40.88 | 1.05 | 2.05 | 95.24 |
| This work  [PM] |  | Commercial membrane + γ-MnO_2_ with FTDS fluorinated. | 5 | 60-20 | 0.477 | 0.616 | 29.14 | 1.05 | 1.30 | 23.81 |

## **Section S17. The analysis of economic prospects.**

To highlight the economic potential of the novel DCMD approach proposed in this work, the Unit Production Cost (*UPC*) of both laboratory-scale and pilot-scale DCMD systems over one-year period are estimated. The *UPC* is defined as follows^[26]^:

$UCP=\frac{C_{T}}{P_{W}}$ (S29)

where *P_W_* (m^3^) is the yearly freshwater production and *C_T_* ($) is the total annual cost. *C_T_* is composed of three components: the annualized capital cost (*C_C_*), the fixed operational cost (*C_F_*) and the variable operational cost (*C_V_*), represented as follows:

$C_{T}=C_{C}+C_{F}+C_{V}$ (S30)

The annualized capital cost (*C_C_*) is estimated via the following equations:

$C_{C}=C_{I} \cdot\alpha$ (S31)

$\alpha=\frac{i\cdot(1+i)^{n}}{(1+i)^{n}-1}$ (S32)

$C_{I}=C_{Eq} +C_{Eng}$ (S33)

where *C_I_* ($) is the initial investment cost. *α* is the amortization factor, which distributes the investment cost fairly over the full operational life. *i* is the interest rate. *n* is the full operational life. *C_Eq_* ($) is the equipment cost, which includes the MD module (membrane and module materials) and associated devices (heat exchanger, instrumentation, pump, etc.). The unit cost of these devices is approximately 150 $ m^-2^ (per membrane area)^[27]^. *C_Eng_* ($) is the engineering cost, at 20% of *C_Eq_*.

The fixed operational cost (*C_F_*) is estimated via the following equations:

$C_{F}=C_{SL}+C_{RMD}$ (S34)

$C_{RMD}=C_{MD}\cdot RF$ (S35)

where *C_SL_* ($) is the service and labor cost, at 2% of *C_C_*. *C_RMD_* ($) is the replacement cost; *C_MD_* ($) is the materials cost of membrane and MD module; *RF* is the annual replacement frequency.

The variable operational cost (*C_V_*) is expressed as:

$C_{V}=C_{E}+C_{Ch}+C_{H}$ (S36)

where *C_E_ ($)* is the electricity cost, *C_Ch_ ($)* is the chemical cost and *C_H_ ($)* is the heat cost.

Based on the membrane fabrication method and the DCMD performances (freshwater productivity and membrane durability) in this work, we estimate the unit MD module cost and the *UPC* for a pilot-scale application (24 m^3^ d^-1^), as shown in **Table S5** and **Table S6**. Generally, pilot-scale MD module is non-detachable, and the module materials cost is significantly higher than membrane cost. Thus, the proportion of increased cost associated with membrane modifications is only 1.8%. The capital cost and operational energy consumption data are provided by a professional MD equipment manufacturer, and the cost of MD module accounts for approximately 60% of the initial investment^[28]^. Compared to the conventional DCMD (demoted as DCMD_conv) approach, the novel approach, DCMD_bub, requires a smaller membrane area and a lower MD replacement frequency, leading to reductions in initial investment and replacement cost by approximately 16.8% and 58.4%, respectively. The operation cost is sensitive to the energy price due to a high energy consumption. When using waste, solar, or steam as heat sources, the *UPC* of the novel approach (with residual H_2_O_2_) is reduced by approximately 40.2%, 25.2%, and 20.4%, respectively.

**Table S5.** Unit MD module cost of pilot-scale systems.

| Cost Composition | | | | | | | | | | | |
| --- | --- | --- | --- | --- | --- | --- | --- | --- | --- | --- | --- |
|  | Unit Price | | | | | Amount | | | | Cost  ($ m^-2^) | Cost increase (%) |
| Materials | PVDF ($ m^-2^) | MnO_2_ ($ g^-1^) | FDTS ($ mL^-1^) | Nafion ($ mL^-1^) | Module ($ m^-2^) | PVDF (m^2^) | MnO_2_ (g m^-2^) | FDTS (mL m^-2^) | Nafion (mL m^-2^) |  |  |
| Pristine membrane | 150 | 0.0 | 0.0 | 0.0 | 250 | 1 | 0 | 0 | 0 | 400 | — |
| Modified membrane | 150 | 0.0021 | 0.70 | 1.4 | 250 | 1 | 2.264 | 3.396 | 3.396 | 407 | 1.75 |

**Table S6.** Unit Production Cost (*UPC*) of pilot-scale systems (24 m^3^ d).

| Factors | | | | | | | | | | | | |
| --- | --- | --- | --- | --- | --- | --- | --- | --- | --- | --- | --- | --- |
| Interest rate (%) | Full operational life (year) | | Heat recovery (%) | | Replacement frequency (times year^-1^) ^a*^ | | Annual operating hours  (h) | | Annual productivity  (m^3^) | | Electricity consumption  (kWh m^-3^) ^b*^ | |
| 5 | 20 | | 90 | | 0.1/0.2 | | 24×365 | | 8760 | | 2 | |
| Cost Composition | | | | | | | | | | | | |
| Cost ($) | | Waste heat | | | | Solar heat ^c*^ | | | | Industrial steam ^d*^ | | |
|  |  | Heat consumption: — | | | | Heat consumption ^e*^:150 (kWh m^-3^) | | | | Heat consumption ^f*^:0.2 (ton m^-3^) | | |
|  |  | DCMD_conv | | DCMD_bub | | DCMD_conv | | DCMD_bub | | DCMD_conv | | DCMD_bub |
| Annual capital (C_C_) | | 23673.29 | | 19701.26 | | 23673.29 | | 19701.26 | | 23673.29 | | 19701.26 |
| Equipment (C_Eq_) ^g*^ | | 245850 | | 204600 | | 245850 | | 204600 | | 245850 | | 204600 |
| Engineering C_Eng_) | | 49170 | | 40920 | | 49170 | | 40920 | | 49170 | | 40920 |
| Fixed operational (C_F_) | | 36233.47 | | 15274.03 | | 36233.47 | | 15274.03 | | 36233.47 | | 15274.03 |
| Service & labor (C_SL_) | | 473.47 | | 394.03 | | 473.47 | | 394.03 | | 473.47 | | 394.03 |
| Replacement (C_RMD_) | | 35760 | | 14880 | | 35760 | | 14880 | | 35760 | | 14880 |
| Variable operational (C_V_) | | 2058.6 | | 2058.6 (20454.6) | | 38850.6 | | 38850.6 (57246.6) | | 62502.6 | | 62502.6 (80898.6) |
| Chemical (C_Ch_) ^h*^ | | 131.4 | | 131.4 (18527.4) | | 131.4 | | 131.4 (18527.4) | | 131.4 | | 131.4 (18527.4) |
| Electricity (C_E_) | | 1927.2 | | 1927.2 | | 1927.2 | | 1927.2 | | 1927.2 | | 1927.2 |
| Heat (C_H_) | | 0 | | 0 | | 36792 | | 36792 | | 60444 | | 60444 |
| Total (C_T_) | | 61965.36 | | 37033.89 (55429.89) | | 98757.36 | | 73825.89 (92221.89) | | 122409.36 | | 97477.89 (115873.89) |
| *UPC* ($ m^-3^) | | 7.07 | | 4.23 (6.33) | | 11.27 | | 8.43 (10.53) | | 13.97 | | 11.13 (13.23) |
| Cost reduction (%) | | — | | 40.2 (10.51) | | — | | 25.22 (6.6) | | — | | 20.35 (5.32) |

Note: a*: According to the experimental results presented in **Section S16**, the antifouling performance of modified membrane is twice that of the pristine membrane, resulting in a 50% reduction in replacement frequency.

b* - d*: The electricity consumption of the system comes from components such as pumps, controls, and sensors. The price is assumed to be 0.11 $ kWh^-1^. The solar heat price and the industrial steam price are assumed to be 0.028 $ kWh^-1[29]^ and 34.5 $ ton^-1[30]^, respectively.

e* & f*: The pilot-scale plant is assumed to be a multi-stage DCMD system consisting of seven stages, with a temperature difference of 5°C between consecutive stages. The feed and permeate side inlet temperatures of the membrane module are set at 60°C and 20°C, respectively, and the flow velocity is 5 m min^-1^. Therefore, the calculated heat recovery rate is approximately 90% (the terminal temperature difference is 60°C/55°C), and the unit heat consumption is 150 kWh m^-3^ (0.2 ton m^-3^).

g*: According to the experimental results in this work, the productivities of the modified membrane and the pristine membrane are 3.23 (LMH) and 2.69 (LMH), respectively. Therefore, the calculated areas of MD modules are approximately 372 m^2^ and 447 m^2^.

h*: The most chemical consumable for DCMD_bub is H_2_O_2_. Based on the experimental results and the reported catalytic reaction rates^[31]^, we estimate the H_2_O_2_ consumption is 4500 (g h^-1^) at the pilot-scale system, accounting for an ineffective loss of 50%. The price of H_2_O_2_ (30 wt%) is 140 ($ ton^-1^), as provided by the chemical plant. Therefore, the calculated cost of H_2_O_2_ is 2.1 $ m^-3^. When utilizing the residual H_2_O_2_ in the wastewater, the cost is assumed to be zero. Additionally, the cost of other conventional chemicals consumption is assumed to be 0.015 $ m^-3^.

## **Section S18. The mechanism of catalytic H_2_O_2_ decomposition by MnO_2_.**

The possible decomposition reaction of H_2_O_2_ catalyzed by MnO_2_, involving the reversible transition of manganese ions, is detailed in Equations S37-S39:

 (S37)

$Mn^{4+}+O_{2}\xrightarrow{}Mn^{3+}+{}^{1}O_{2}$ (S38)

 (S39)

To determine the appropriate dosage for our experimental conditions, we conducted preliminary flux tests across a range of H_2_O_2_ concentration from 0.1 to 1 wt%, which is relevant to practical applications. As shown in **Figure S12**, the flux initially increased but then decreased with higher concentrations, indicating that excessive H_2_O_2_ impaired performance. This phenomenon could be attributed to intensified bubble generation at elevated H_2_O_2_ levels, which enhances turbulence while simultaneously hindering mass transfer due to gas permeation. Consequently, the minimum effective H_2_O_2_ concentration (0.1 wt%) was selected for subsequent experiments.

Under practical conditions, the endogenous H_2_O_2_ in wastewater proved sufficient without supplementation. However, at laboratory scale, we performed the following calculations to ensure that the determined dosage would sufficient for sustained bubble generation throughout the experiments: (i) Firstly, the effective volume of the membrane module was 3.92 mL (49 mm × 40 mm × 2 mm), and the total MnO_2_ content on the membrane surface was measured as 4.44 mg (2.264 g m^-2^), resulting in a calculated MnO_2_ concentration of 0.013 mol L^-1^. (ii) Secondly, at the initial concentration of 0.1 wt%, the total H_2_O_2_ in the feed tank was 0.088 mol. Assuming 50% spontaneous decomposition of at 60 °C, the effective H_2_O_2_ concentration in the feed liquid was 0.015 mol L^-1^. (iii) Thirdly, the ratio of [H_2_O_2_] / [≡MnO_2_] was calculated to be 1.136. Based on reported catalytic reaction rates^[31, 32]^, the observed reaction rate constant (*k_obs_*) is approximately 15 min⁻¹, suggesting that H_2_O_2_ depletion in the module would require about 16 seconds. (iv) Finally, on the basis of above, complete consumption of H_2_O_2_ in the feed tank would take approximately 5.5 hours. Overall, these results confirm that the initial H_2_O_2_ dosage (0.1 wt%) significantly exceeded operational demands, eliminating the need for supplementation during an experimental cycle.


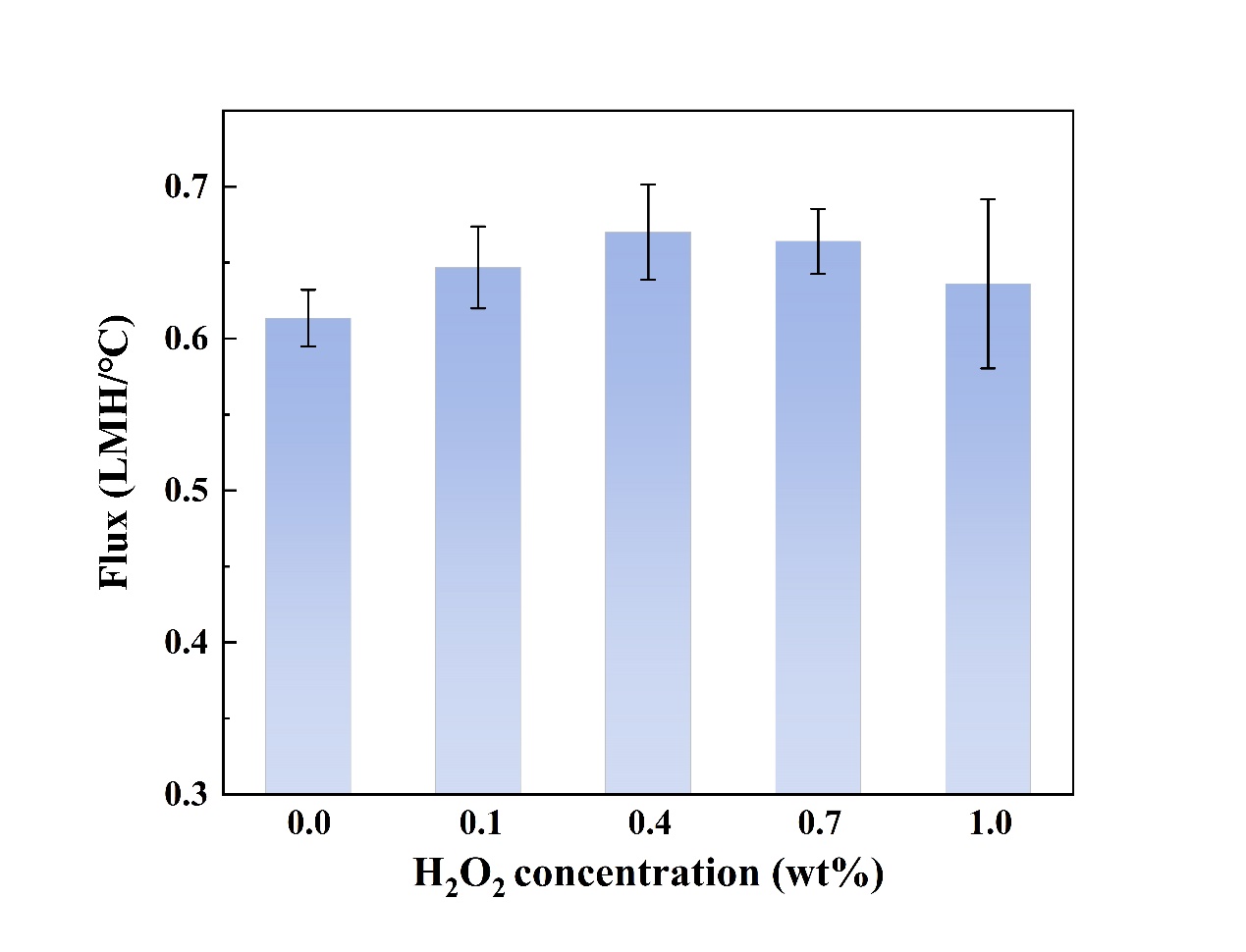


**Figure S12.** **The flux under different H_2_O_2_ concentrations.**

## **SI References**

[1] G. Fan, C. Chen, X. Chen, Z. Li, S. Bao, J. Luo, D. Tang, Z. Yan, *Sci. Total Environ.* **2021**, *801*, 149611.

[2] E. Hayashi, Y. Yamaguchi, K. Kamata, N. Tsunoda, Y. Kumagai, F. Oba, M. Hara, *J. Am. Chem. Soc.* **2019**, *141*, 18642.

[3] I. O. Arukalam, E. E. Oguzie, Y. Li, *J. Colloid. Interface. Sci.* **2016**, *484*, 220.

[4] K. A. Lin, W. D. Oh, M. W. Zheng, E. Kwon, J. Lee, J. Y. Lin, X. Duan, F. Ghanbari, *J. Colloid. Interface. Sci.* **2021**, *592*, 416.

[5] J. Cech, R. Taboryski, *Appl. Surf. Sci.* **2012**, *259*, 538.

[6] S. Xie, Z. Pang, C. Hou, N. H. Wong, J. Sunarso, Y. Peng, *J. Membr. Sci.* **2022**, 660, 120846.

[7] B. Zhang, J. Shen, H. Tang, Y. Shen, J. Li, Z. Zhu, *Desalination* **2022**, 539, 115969.

[8] B. Sun, M. Wu, H. Zhen, Y. Jia, P. Li, Z. Yuan, X. Li, G. He, X. Jiang, *Desalination* **2024**, 573, 117177.

[9] J. Zhou, Y. Li, Q. Wu, Z. Liu, M. Zhang, H. Hu, X. Wang, X. Lu, C. Wu, *Sep. Purif. Technol.* **2025**, 364, 132449.

[10] H. F. Juybari, M. Karimi, R. Srivastava, J. Swaminathan, D. M. Warsinger, *Desalination* **2023**, 553, 116411.

[11] Z. Ma, X. Chen, M. Jia, H. Mao, M. Li, S. Zhou, J. H. Xin, Y. Zhao, *Sep. Purif. Technol.* **2024**, 341, 126861.

[12] Z. Ma, X. Chen, H. Mao, D. Zhang, S. Zhou, M. Li, Y. Zhao, W. Peng, J. H. Xin, *Desalination* **2024**, 580, 117547.

[13] Y. M. Manawi, M. A. M. M. Khraisheh, A. K. Fard, F. Benyahia, S. Adham, *Desalination* **2014**, *341*, 38.

[14] K. G. Nayar, M. H. Sharqawy, L. D. Banchik, J. H. Lienhard V, *Desalination* **2016**, *390*, 1.

[15] Q. Chen, M. Kum Ja, Y. Li, K. J. Chua, *Appl. Energy* **2018**, *230*, 960.

[16] P. A. B. de Sampaio, *Desalination* **2022**, *533*, 115769.

[17] I. Hitsov, T. Maere, K. De Sitter, C. Dotremont, I. Nopens, *Sep. Purif. Technol.* **2015**, *142*, 48.

[18] M. Khayet, *Adv. Colloid. Interface. Sci.* **2011**, *164*, 56.

[19] A. Khalifa, H. Ahmad, M. Antar, T. Laoui, M. Khayet, *Desalination* **2017**, *404*, 22.

[20] A. Alkhudhiri, N. Darwish, N. Hilal, *Desalination* **2012**, *287*, 2.

[21] Y. Kim, H. Lee, G. Gil, H. Ji, Y. Kim, *Water Res.* **2023**, *229*, 119407.

[22] G. Dong, J. F. Kim, J. H. Kim, E. Drioli, Y. M. Lee, *Desalination* **2017**, *402*, 72.

[23] H. J. Hwang, K. He, S. Gray, J. Zhang, I. S. Moon, *J. Membr. Sci.* **2011**, *371*, 90.

[24] R. D. Gustafson, J. R. Murphy, A. Achilli, *Desalination* **2016**, *378*, 14.

[25] T. Horseman, Y. Yin, K. S. Christie, Z. Wang, T. Tong, S. Lin, *Acs ES&T Eng.* **2021**, *1*, 117.

[26] M. A. Jamil, S. M. Elmutasim, S. M. Zubair, *Energy Conv. Manag.* **2018**, *158*, 286.

[27] S. M. Alawad, D. U. Lawal, A. E. Khalifa, I. H. Aljundi, M. A. Antar, T. N. Baroud, M. A. M. Eltoum, *Desalination* **2023**, *566*, 116894.

[28] R. Schwantes, K. Chavan, D. Winter, C. Felsmann, J. Pfafferott, *Desalination* **2018**, *428*, 50.

[29] J. Yan, Y. Yang, P. Elia Campana, J. He, *Nat. Energy* **2019**, *4*, 709.

[30] W. Zhong, Z. Dai, X. Lin, G. Pan, *Energy* **2024**, *296*, 131056.

[31] S. Do, B. Batchelor, H. Lee, S. Kong, *Chemosphere* **2009**, *75*, 8.

[32] S. Lin, M. D. Gurol, *Environ. Sci. Technol.* **1998**, 32, 1417.
